# Mechanosensitive PIEZO2 channels shape coronary artery development

**DOI:** 10.1038/s44161-025-00677-3

**Published:** 2025-06-27

**Authors:** Mireia Pampols-Perez, Carina Fürst, Oscar Sánchez-Carranza, Elena Cano, Jonathan Alexis Garcia-Contreras, Lisa Mais, Wenhan Luo, Sandra Raimundo, Eric L. Lindberg, Martin Taube, Arnd Heuser, Anje Sporbert, Dagmar Kainmueller, Miguel O. Bernabeu, Norbert Hübner, Holger Gerhardt, Gary R. Lewin, Annette Hammes

**Affiliations:** 1 Molecular Pathways in Cortical Development, Max Delbrück Center for Molecular Medicine in the Helmholtz Association (MDC), Berlin, Germany; 2 Institute for Biology, Free University of Berlin, Berlin, Germany; 3 Molecular Physiology of Somatic Sensation Laboratory, Max Delbrück Center for Molecular Medicine in the Helmholtz Association (MDC), Berlin, Germany; 4 Integrative Vascular Biology, Max Delbrück Center for Molecular Medicine in the Helmholtz Association (MDC), Berlin, Germany; 5 German Center for Cardiovascular Research (DZHK), partner site Berlin, Berlin, Germany; 6 Area of Human Anatomy and Embryology, Faculty of Medicine, University of Malaga, Málaga, Spain; 7 Biomedical Image Analysis, Max-Delbrück-Center for Molecular Medicine in the Helmholtz Association (MDC), Berlin, Germany; 8 Helmholtz Imaging, Berlin, Germany; 9 Digital Engineering Faculty, University of Potsdam, Potsdam, Germany; 10 Advanced Light Microscopy & Image Analysis, Max Delbrück Center for Molecular Medicine in the Helmholtz Association (MDC), Berlin, Germany; 11 Cardiovascular and Metabolic Sciences, Max Delbrück Center for Molecular Medicine in the Helmholtz Association (MDC), Berlin, Germany; 12 Animal Phenotyping, Max Delbrück Center for Molecular Medicine in the Helmholtz Association (MDC), Berlin, Germany; 13 Centre for Medical Informatics, Usher Institute, The University of Edinburgh, Edinburgh, UK; 14 Charité-Universitätsmedizin Berlin, Berlin, Germany; 15 Helmholtz Institute for Translational AngioCardiosciences (HI-TAC), Max Delbrück Center for Molecular Medicine at Heidelberg University, Heidelberg, Germany; 16 German Center for Mental Health (DZPG), partner site Berlin, Berlin, Germany

**Keywords:** Cell lineage, Genetics

## Abstract

Coronary arteries develop under constant mechanical stress. However, the role of mechanosensitive ion channels in this process remains poorly understood. Here we show that the ion channel PIEZO2, which responds to mechanical stimuli, is expressed in specific coronary endothelial cell populations during a critical phase of coronary vasculature remodeling. These *Piezo2*^+^ coronary endothelial cells show distinct transcriptional profiles and have mechanically activated ionic currents. Strikingly, PIEZO2 loss-of-function mouse embryos and mice with human pathogenic variants of *PIEZO2* show abnormal coronary vessel development and cardiac left ventricular hyperplasia. We conclude that an optimal balance of PIEZO2 channel function contributes to proper coronary vessel formation, structural integrity and remodeling, and is likely to support normal cardiac function. Our study highlights the importance of mechanical cues in cardiovascular development and suggests that defects in this mechanosensing pathway may contribute to congenital heart conditions.

## Abstract (web summary)

Pampols-Perez et al. identify the mechanosensitive ion channel PIEZO2 as a novel marker for embryonic coronary artery endothelial cells and as a critical regulator of coronary vascular remodeling. They show that in a distinct subset of coronary endothelial cells, PIEZO2 translates mechanically activated ionic currents into biological signals guiding coronary artery morphogenesis.

## Main

The branching system of the coronary vasculature has a unique structure adapted to the particular physiology of the heart muscle. During its formation in the embryo and throughout adult life, the coronary vasculature is uniquely challenged by mechanical loads from the heartbeat more than 100,000 times a day and from coronary perfusion pressure. The coronary arteries are particularly susceptible to disease. Pathologies of these vessels are a main cause of ischemic heart disease, a leading cause of mortality in Western societies^1^. In this context, a better understanding of embryonic coronary artery formation processes holds the potential to recreate and enhance the developmental process for coronary artery regeneration under disease conditions.

Embryonic coronary vasculature formation involves the intricate orchestration of endothelial cell populations, originating from three primary sources: the sinus venosus (SV), the endocardium and the (pro)epicardium^2^. While the role of chemotaxis in guiding angiogenesis is well described, also for coronary vasculature^3,4^, there is emerging evidence that mechanical guidance cues, termed durotaxis, have a role in the patterning and morphogenesis of blood vessels^5^. However, there are only few reports on the presence and function of mechanosensitive ion channels in endothelial cells during vasculogenesis or angiogenesis^6^. In particular, piezo-type mechanosensitive ion channel component 1 (PIEZO1), which is widely expressed in many cell types^7^, is known to play an important role in the initiation of embryonic vessel formation and function^8,9^. PIEZO1-deficient mice show disrupted vascularization and a failure in the maturation of endothelial cells into larger blood vessels^9,10^. The role of its homolog, piezo-type mechanosensitive ion channel component 2 (PIEZO2) in angiogenesis during embryonic development has so far not been explored. However, expression of *Piezo2* in endothelial cells of the brain and lungs has been suggested before^8,11^. One study reported PIEZO2 to be involved in tumor angiogenesis^12^, and another study described a dramatic loss of pulmonary microvascular endothelial cells in endothelial cell-specific *Piezo2* knockout rats^13^.

Ever since the discovery of mechanosensitive PIEZO channels^7^, PIEZO2 has been primarily characterized as a sensory ion channel, present in a variety of sensory neuron types, the function of which is to sense mechanical forces important for touch, interoception and pain^14,15^. Importantly, *PIEZO2* pathogenic variants are associated with congenital disorders in humans, including joint, craniofacial, brain and cardiovascular defects^16,17^. Interestingly, the University of Cambridge PhenoScanner V2 database of human genotype–phenotype associations suggests a PIEZO2 genetic linkage with heart failure, diastolic blood pressure, hypertensive heart disease and thoracic aortic aneurysm^18^. However, the precise function of mechanosensitive PIEZO2 channels in non-sensory cells, including endothelial cells, remains largely unstudied. In particular, a specific function of mechanosensitive ion channels in coronary artery endothelial cells during embryonic development has not been studied. Here we aimed to investigate the role of PIEZO2 in the developing cardiovascular system. Using a combination of genetic fate mapping, single-cell sequencing, physiological analysis and light-sheet imaging, our study uncovers a unique role of PIEZO2 channels in shaping the coronary vasculature during development. Specifically, we show that dysfunction or loss of PIEZO2 leads to aberrant coronary artery branching, impaired vessel morphology and cardiac hyperplasia.

## Results

### Fate mapping PIEZO2 cells in the embryonic heart

**Fig. 1:**
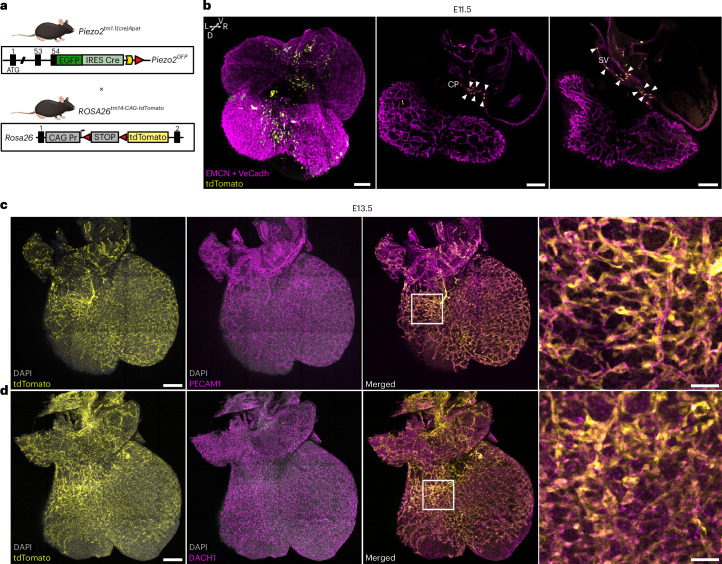
*Piezo2*-driven tdTomato fate mapping in the developing heart. **a**, Outline of the genetic strategy (generated in affinity designer 2) used to generate the *Piezo2*-driven tdTomato fate mapping model. **b**, A 3D rendering of a representative E11.5 tdTomato^+^ heart with all cardiac endothelial cells immunolabeled with a cocktail of anti-VE-cadherin (VeCadh) and anti-EMCN antibodies (magenta). The tdTomato signal (yellow) was found in the SV and the nascent CP (white arrows in the optical sections) (scale bars, 100 µm) (*n* = 2). **c**, A 3D rendering of a representative E13.5 tdTomato^+^ heart confirming the endothelial fate (PECAM1 positive, magenta) of tdTomato^+^ (yellow) cells in the nascent CP (scale bars, 200 µm and 50 µm in magnification) (*n* = 2). **d**, A 3D rendering of a representative E13.5 tdTomato^+^ heart immunolabeled against the coronary marker DACH1. The co-localization of the tdTomato^+^ cells (yellow) with DACH1 (magenta) shows that *Piezo2* fate-mapped cells were coronary endothelial cells (scale bars, 200 µm and 25 µm in magnification) (*n* = 2).

**Fig. 2:**
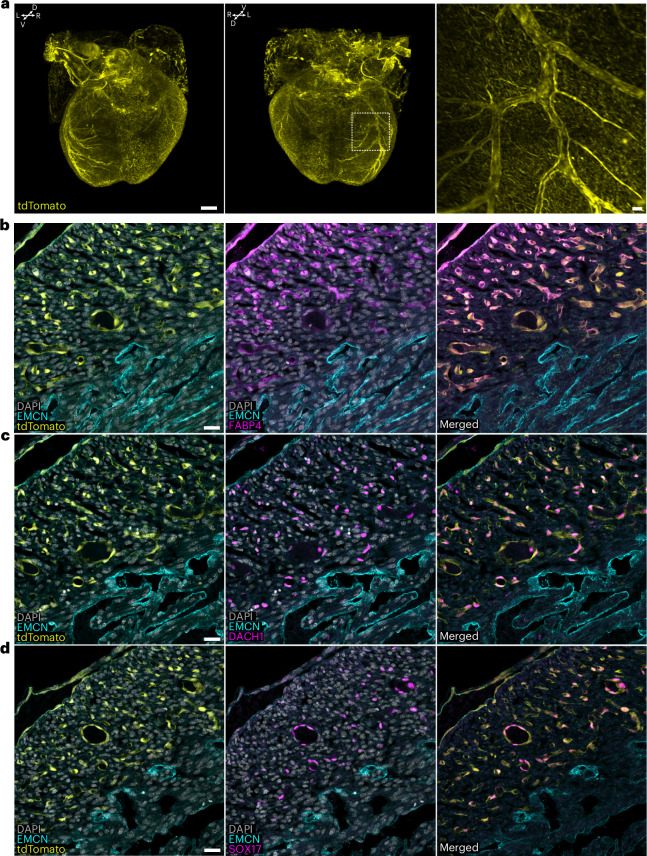
*Piezo2*-driven fate mapping sharply defines the coronary vasculature. **a**, A 3D rendering of a representative E18.5 heart showing that tdTomato^+^ (yellow) cells contribute to the development of the coronary vasculature (scale bars, 300 µm (3D) and 20 µm (zoom in) (*n* = 4)). **b**–**d**, E18.5 tdTomato^+^ hearts were immunolabeled for EMCN (transversal sections; EMCN, cyan) and three different coronary markers: FABP4 (magenta) (**b**), DACH1 (magenta) (**c**) and SOX17 (magenta) (**d**). Immunolabeling revealed that tdTomato^+^ cells comprise coronary endothelial cells of small (DACH1 positive) and mature (SOX17 positive) arteries, as well as endothelial cells in the coronary FABP4-positive population. tdTomato signals did not co-localize with EMCN in the endocardium (cyan), confirming the coronary endothelial cell nature of the tdTomato^+^ cells (scale bars, 25 µm) (*n* = 2).

**Extended Data Fig. 1:**
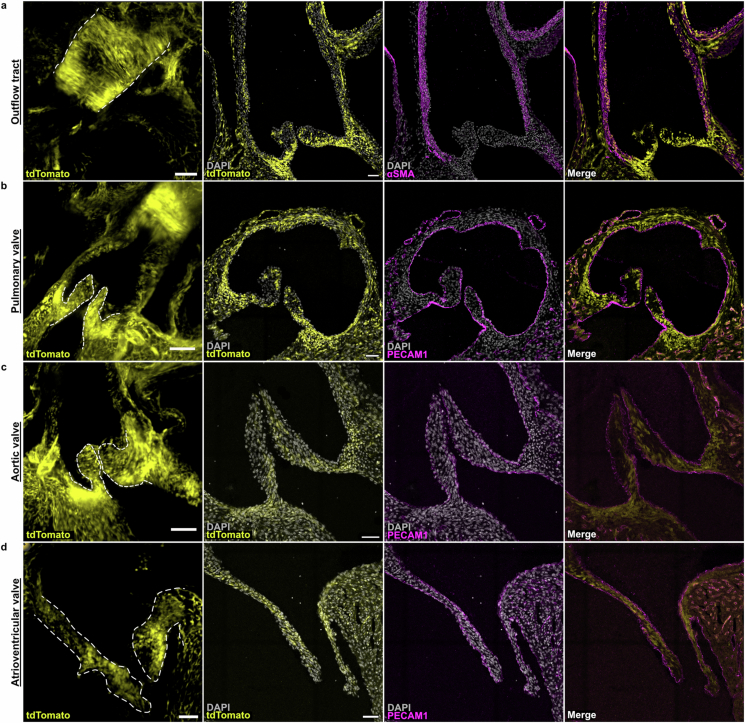
Piezo2-driven tdTomato is expressed in the outflow tract and the cardiac valves. **a - d**, Outflow tract and cardiac valves three-dimensional rendering and immunolabelled sections from an E18.5 tdTomato^+^ heart. tdTomato signal (yellow) in the outflow tract (**a**) colocalizes with αSMA (magenta). In the cardiac valves (**b - d**), tdTomato+ cells (yellow) are surrounded by endothelial cells (PECAM1 positive, magenta), indicating that they are likely valve interstitial cells (n = 2, Scale bar: 100 µm [3D] and 50 µm [sections]).

To delineate the trajectory and contribution of *Piezo2*^+^ cells in the mouse embryonic heart, we used a genetic fate mapping approach in which *Piezo2-Cre* (ref. ^15^) drives *tdTomato* expression in *Piezo2-*expressing cells and their progeny (*Piezo2-tdTomato* mice) (Fig. 1a). Whole-mount three-dimensional (3D) confocal imaging revealed specific populations of tdTomato-positive (tdTomato^+^) cells in the heart at embryonic day 11.5 (E11.5) (Fig. 1b). We found tdTomato^+^ cells in the SV and the nascent coronary plexus (CP), and we identified them as endothelial cells positive for endomucin (EMCN) and vascular endothelial cadherin (VE-cadherin) (Fig. 1b, arrowheads). At E13.5, the tdTomato^+^ cell population had expanded to form a nascent coronary endothelial plexus as evidenced by cells staining positive for the pan-endothelial marker platelet/endothelial cell adhesion molecule 1 (PECAM1) (Fig. 1c). Whole-mount confocal microscopy also revealed that tdTomato^+^ cells were positive for Dachshund homolog 1 (DACH1), a transcription factor specifically expressed in embryonic coronary endothelial cells^3,19^ (Fig. 1d). Thus, the *Piezo2-*expressing cell lineage specifically contributes to the developing coronary vasculature. At E13.5, the coronary plexus finally connects to the ascending aorta and triggers plexus remodeling into a mature vasculature. At E18.5, tdTomato^+^ cells now clearly form the neonatal coronary vasculature as revealed by light-sheet microscopy imaging of the entire heart (Fig. 2a and Supplementary Video 1). Optical sections through the whole-mount light-sheet images highlighted the connection of the tdTomato^+^ fate-mapped vessels to the ascending aorta, indicating that these vessels are the coronary arteries (Supplementary Video 2). The coronary vessel character was verified on transverse cryosections, and we identified tdTomato^+^ cells as coronary endothelial cells by fatty acid binding protein 4 (FABP4) staining^20^ (Fig. 2b). Further characterization showed that tdTomato^+^ cells contribute to both small arteries in the remodeling zone (DACH1 positive)^3^ (Fig. 2c) and mature arteries, which are positive for SRY-box transcription factor 17 (SOX17)^21^ (Fig. 2d). EMCN-positive cells in the endocardium were negative for tdTomato signals (Fig. 2b–d), supporting the coronary nature of *Piezo2* fate-mapped endothelial cells. We observed other non-endothelial cell types with tdTomato labeling, one in the outflow tract with morphological characteristics of vascular smooth muscle cells. These cells were also positive for alpha-smooth muscle actin (αSMA), confirming their identity as vascular smooth muscle cells. Cells making up all four valves of the heart were also tdTomato^+^ and were surrounded by endothelial cells, indicating their likely identity as valve interstitial cells (Extended Data Fig. 1). As *Piezo2*-lineage endothelial cells evidently contribute to coronary vasculature formation, we proceeded to analyze both, *Piezo2* expression and the transcriptional profile of *Piezo2-*expressing cells.

### *Piezo2* expression in distinct cardiac endothelial cell types

**Fig. 3:**
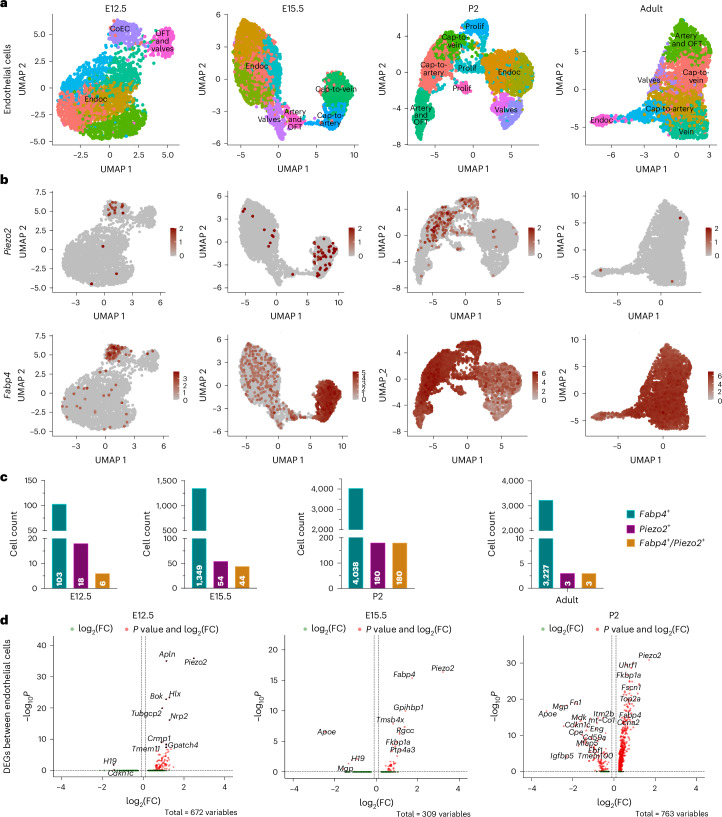
scRNA-seq confirmed ongoing expression of *Piezo2* in the developing coronary endothelial cells. **a**, Uniform manifold approximation and projection of cECs from E12.5, E15.5, P2 and murine adult hearts (8 weeks old) showing the following clusters: endocardium (Endoc), capillary-to-vein (Cap-to-vein), capillary-to-artery (Cap-to-artery), proliferating cells (Prolif), valvular endocardium (Valves), arterial and outflow tract cells (Artery and OFT) and venous cells (Vein). **b**, Representation of *Piezo2* and *Fabp4* expression in various clusters of cECs. **c**, *Fabp4*^+^, *Piezo2*^*+*^ and *Fabp4*^+^*/Piezo2*^*+*^ cell counts at E12.5, E15.5, P2 and adult. Color scale bars indicate log-normalized expression. **d**, Volcano plots showing DEGs between individual *Piezo2-*expressing and *Piezo2* non-expressing endothelial cells. DEGs for each cluster were identified using the FindAllMarkers function in Seurat, applying the Wilcoxon rank sum test (two sided). Genes were considered for analysis if expressed in at least 25% of cells in either cluster and showed a log_2_(fold change (FC)) of at least 0.25. Genes with an adjusted *P* value less than 0.05 were classified as DEGs. Source data

**Extended Data Fig. 2:**
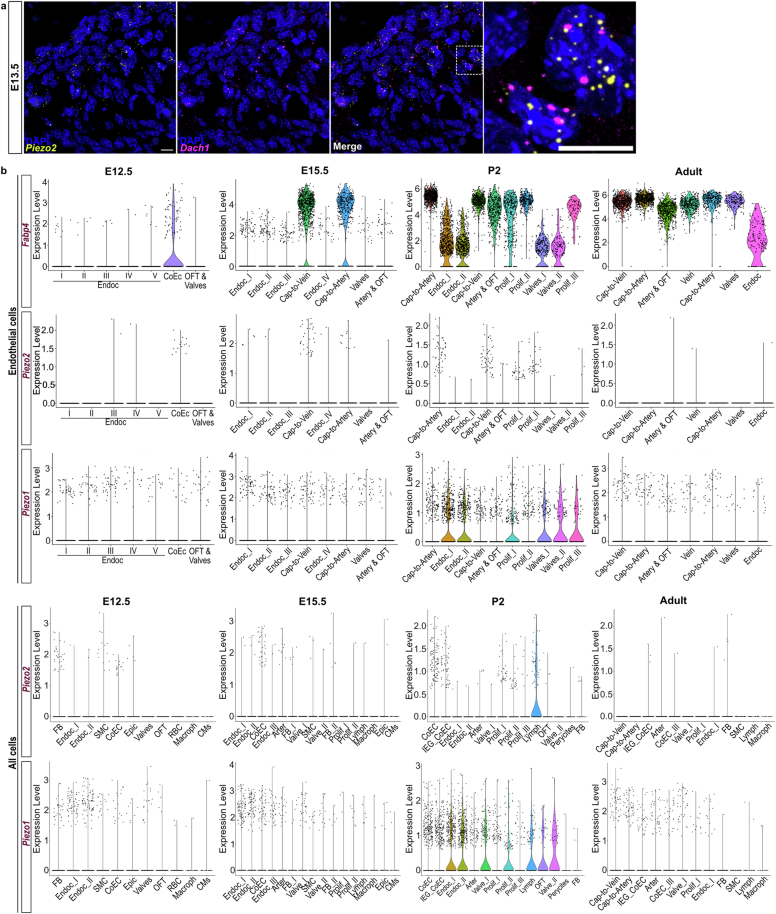
Expression of Piezo2 mechanosensitive ion channels in heart tissue. **a**, Expression of *Piezo2* mechanosensitive ion channels in heart tissue. Single-molecule in situ hybridization showing coronary endothelial cells expressing *Piezo2* and *Dach1* at E13.5. Nuclei are labeled with DAPI. Scale bar: 10 µm. Hybridization was performed on at least 5 sections and repeated on 2 different hearts. **b**, scRNAseq identifies distinct cluster representations for *Piezo2*^*+*^ and *Piezo1*^*+*^ cardiac cells. Volcano plots showing *Fabp4*, *Piezo1* and *Piezo2* expression levels on cardiac endothelial cells and all cardiac cells from E12, E15, P2 and murine adult hearts (8 weeks old) showing the following clusters: endocardium (Endoc_I to Endoc_V), coronary endothelium (cap-to-vein, cap-to-artery and CoEC_III), proliferating cells (Prolif_I to Prolif_II), valvular endocardium (Valve_I and Valve_II), arterial cells (Arter), venous cells (Vein), outflow tract cells (OFT), pericytes, fibroblasts (FB_I and FB_II), lymphatic cells (lymph), smooth muscle cells (SMC), epicardial cells (Epic), macrophages (macroph) and red blood cells (RBC), Cardiomyocytes (CMs), Immediate Early Genes-expressing Coronary Endothelium (IEG_CoEC). Note: The analysis includes some non-endothelial cells, as the isolation of CD31 + /CD45- endothelial cells by BD FACSAria II sorting following heart dissociation is never completely pure (Cano et al., 2025, Circ. Res; DOI: 10.1161/CIRCRESAHA.124.324868).

While fate mapping provided insights into the trajectories of *Piezo2-*expressing cells, examining the transcriptional profile of *Piezo2-*expressing cells could help identify the developmental stages in which this mechanosensitive ion channel contributes to coronary development. By using single-molecule fluorescence in situ hybridization, we could show continued *Piezo2* mRNA expression in coronary endothelial cells at E13.5 along with *Dach1* expression (Extended Data Fig. 2a). In addition, single-cell RNA (scRNA) datasets from fluorescence-activated cell sorting-sorted cardiac endothelial fractions (CD31^+^ alias PECAM1^+^/CD45^−^ alias PTPRC^−^) from four different stages: E12.5, E15.5, postnatal day (P) 2 and adult (8 weeks old) were used to monitor ongoing *Piezo2* expression^22^. Unsupervised clustering was performed using the uniform manifold approximation and projection method, and cell type clusters were classified based on the expression profiles of the top differentially expressed genes (DEGs)^22^ (Fig. 3a). At E12.5, *Piezo2* expression was mainly found in the coronary endothelial cells (Fig. 3b). Subsequently, at E15.5, *Piezo2* expression was observed in two subclusters of coronary endothelial cells designated capillary-to-vein and capillary-to-artery, which are capillary cells^22^ (Fig. 3b). At P2, *Piezo2* expression was still present in the same capillary cell types (cap-to-vein and cap-to-artery) and in the proliferating cell cluster (Fig. 3b). However, in adult hearts, no *Piezo2* expression was found in any of the endothelial cell types analyzed (Fig. 3b). To quantify the proportion of *Piezo2*^+^ and *Fabp4*^+^ cells, we extracted data from our scRNA sequencing (scRNA-seq) analysis across different developmental stages (E12.5, E15.5, P2 and adult). The strong correlation between *Piezo2*^+^ and *Fabp4*^+^ endothelial cells across developmental stages (Fig. 3b) suggests that PIEZO2 is specifically enriched in a subset of coronary endothelial cells involved in vascular remodeling. The results show that over the developmental stages, the majority of *Piezo2*^+^ cells become also *Fabp4*^+^ (Fig. 3c). While *Piezo2*^+^/*Fabp4*^+^ cells persist through embryonic and early postnatal stages, *Piezo2* expression is lost in adult endothelial cells (Fig. 3b and Extended Data Fig. 2b). These data show a dynamic *Piezo2* expression pattern in distinctive coronary endothelial cell populations during embryonic heart development. Given that *Fabp4*^+^ cells contribute to capillary-to-artery transitions, the co-expression of *Piezo2* and *Fabp4* indicates that PIEZO2-mediated mechanosensing may play a role in endothelial differentiation and artery formation.

### *Piezo2*^*+*^ coronary cells show distinct gene expression

We next asked whether *Piezo2-*expressing coronary endothelial cells show a distinct transcriptional profile when compared with coronary endothelial cells lacking *Piezo2* expression (Fig. 3d and Supplementary Tables 1–3). At E12.5, *Piezo2*^+^ coronary endothelial cells showed higher expression of genes such as *Neuropilin 2* (*Nrp2*), a receptor for semaphorin family members, and *Apelin* (*Apln*), a sprouting marker, both having been implicated in blood vessel formation^23–25^. We also noticed enrichment of collapsin response mediator protein 1 (Crmp1), a protein involved in neuronal migration and development^26^. *Piezo2* was still enriched at E15.5 along with other well-known markers of coronary endothelial cells such as *Fabp4* (ref. ^27^) and the capillary markers *Rgcc* (regulator of cell cycle) and *Gpihbp1* (glycosylphosphatidylinositol anchored high density lipoprotein binding protein 1)^28^. At P2, *Piezo2*^*+*^ cells still showed enriched expression of *Fabp4* and interestingly were positive for fascin actin-bundling protein 1 (*Fscn1*), an actin-bundling protein that can induce membrane protrusions and is involved in cell migration and motility^29^ (Fig. 3d). The PIEZO2 homolog PIEZO1 is a stretch-activated ion channel that is widely expressed in the cardiovascular system^9,30^. Consistently, our analysis revealed that numerous cell types show *Piezo1* expression across all stages investigated (Extended Data Fig. 2b). By contrast, *Piezo2* expression appeared to be maintained primarily in coronary endothelial cells specifically during the angiogenic phase of heart vascularization. The distinct transcriptional signature of *Piezo2-*expressing coronary endothelial cells, including *Nrp2*, *Apln*, *Crmp1* and *Fscn1*, indicates potential roles in cell migration and motility. These findings provide insights into the potential molecular mechanisms underlying the development and maturation of coronary endothelial cells, highlighting the importance of PIEZO2 and associated markers in cardiovascular biology.

### Endothelial cells show PIEZO2 mechanosensitive currents

**Fig. 4:**
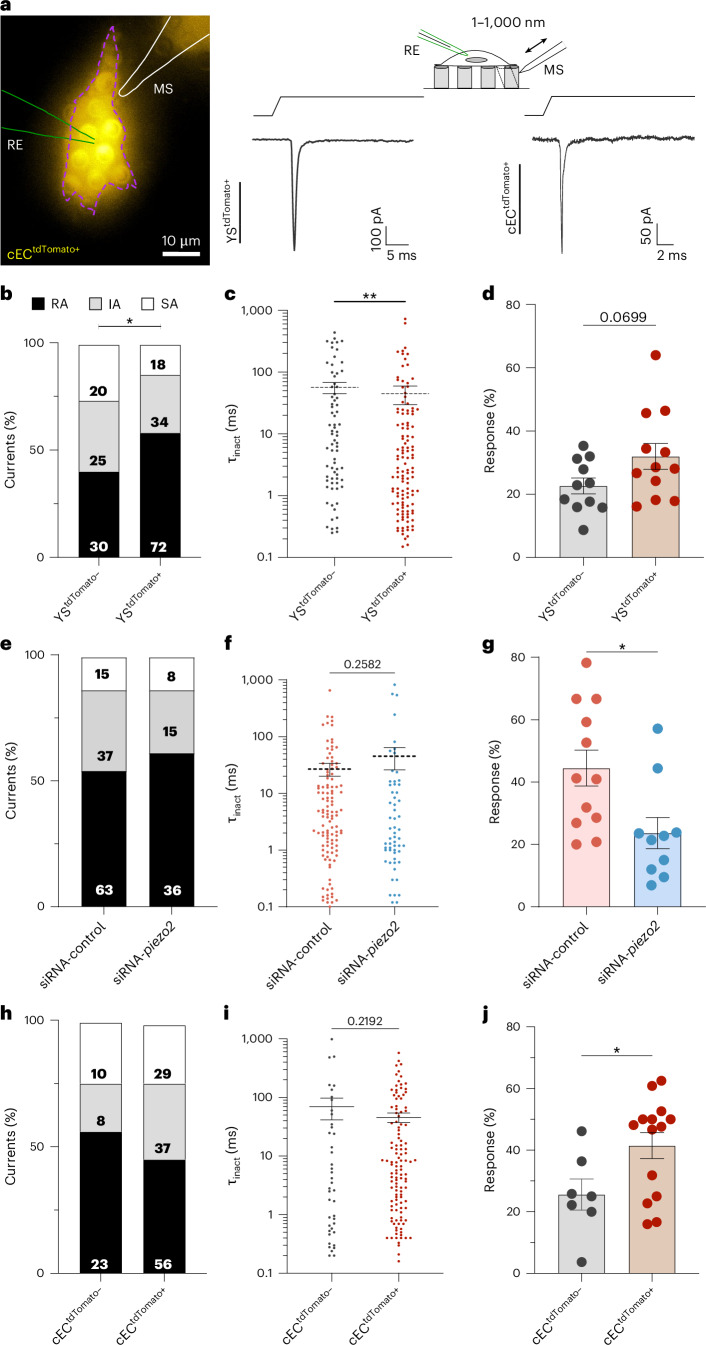
YS cells and cECs showed endogenous deflection-gated currents. **a**, Left: representative picture of a cEC^tdTomato+^ cell cultured on the elastomeric pillar arrays. RE, recording electrode; MS, mechanical stimulator. Right: example traces of RA currents from YS^tdTomato+^ and cEC^tdTomato+^ cells recorded with the pillar array method. **b**, Stacked histograms showing that the proportion of RA currents in YS^tdTomato+^ cells (*n* = 12 biological replicates) was higher than in YS^tdTomato−^ cells (*n* = 11 biological replicates, chi-squared test, two sided, *P* = 0.02). The numbers indicate numbers of currents. **c**, YS^tdTomato+^ cells showed deflection-gated currents with faster inactivation kinetics (τ_inact_ is the time constant of inactivation) compared with non-red cells (Mann–Whitney test, two sided, *P* = 0.004). **d**, YS^tdTomato+^ cells responded statistically similar to YS^tdTomato^^−^ cells, with a trend that shows that they are slightly more sensitive to mechanical stimulation (*t*-test, *P* = 0.0699). **e**,**f**, YS^tdTomato+^ cells did not show differences in the proportion of mechanosensitive currents (**e**) nor changes in inactivation kinetics (**f**) when transfected with *Piezo2*-siRNA. siRNA-control corresponds to cells transfected with non-targeting control siRNA. The numbers indicate numbers of currents. **g**, Plot showing that YS^tdTomato+^ *Piezo2*-siRNA-transfected cells (*n* = 12) responded less to deflection stimuli than control cells (*n* = 10) (Mann–Whitney test, two sided, *P* = 0.01). **h**,**i**, cEC^tdTomato+^ (*n* = 14) and cEC^tdTomato−^ cells (*n* = 7) did not show differences in the proportion of mechanosensitive currents (**h**) nor changes in inactivation kinetics (**i**). The numbers indicate number of currents. **j**, Histogram showing that cEC^tdTomato+^ cells are more responsive to deflection stimuli than cEC^tdTomato−^ cells (Mann–Whitney test, two sided, *P* = 0.0392). Biological replicates are the number of cells, as indicated by *n*. Technical replicates for YS and cEC electrophysiology experiments as indicated in Methods. *P < 0.05; **P < 0.01. All error bars represent standard errors of the mean (±s.e.m.). Source data

**Extended Data Fig. 3:**
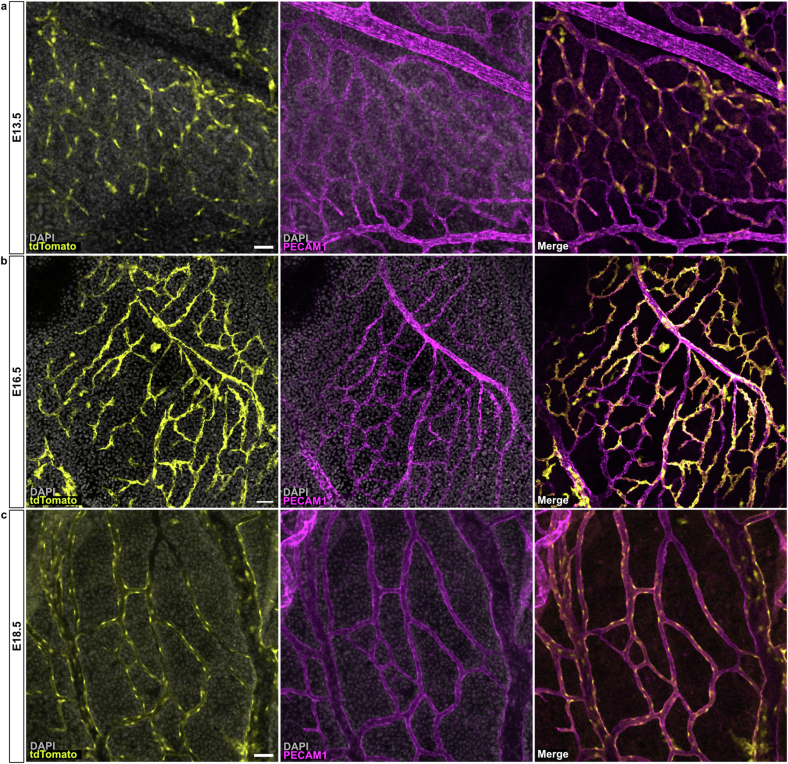
Expression of Piezo2-driven tdTomato in the developing yolk sac. **a - c**, Immunolabeling of whole yolk sacs showing tdTomato signal (in yellow) colocalized with the endothelial marker PECAM1 (in magenta) at embryonic days E13.5 (**a**), E16.5 (**b**), and E18.5 (**c**) (n = 4 per stage, Scale bar: 50 µm).

**Extended Data Fig. 4:**
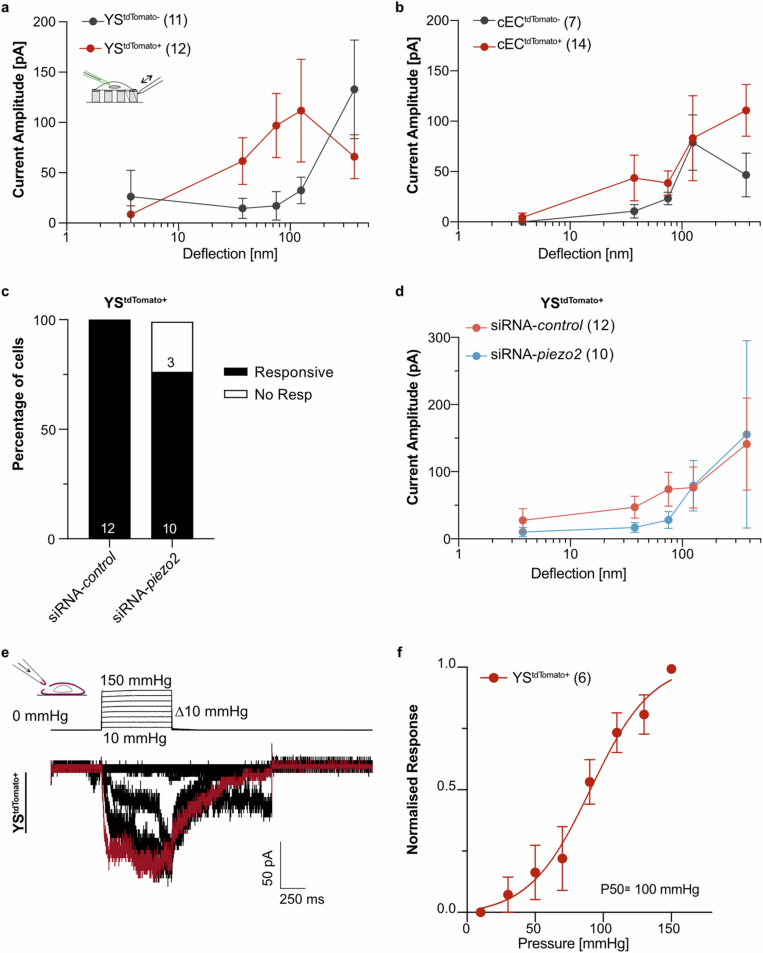
Mechanosensitive currents measured in YSt^dTomato+^ and cEC^tdTomato+^ cells. **a - b**, Deflection-current amplitude relationships of the yolk sac (YS) cells (**a**) and cardiac endothelial cells (cEC) (**b**) showing that deflection-gated currents from tdTomato^+^ and tdTomato^-^ cells displayed similar mechanical responses (Two-way ANOVA, *P* = 0,3025 for YS and p = 0,5806 for cEC). **c**, Stacked histograms showing the percentage of YS^tdTomato+^ either transfected with non-targeting control siRNA or *Piezo2*-siRNA that responded to pillar deflection (Chi-squared test, two-sided, *P* = 0.07). **d**, Deflection-Current amplitude relationship showing similar deflection-responses between YS^tdTomato+^ transfected with control siRNA or *Piezo2*-siRNA. (Two-way ANOVA, *P* = 0,4726). **e**, Representative trace of stretch-sensitive currents stimulated with a pressure-step protocol from outside-out patches in YS^tdTomato+^ cells at a resting membrane potential of -60 mV. **f**, Stretch-response curve of the endogenous stretch-sensitive currents in YS^tdtomato+^ cells. Peak currents were normalized to the maximum current recorded prior to membrane patch rupture. All data is presented as mean ± S.E.M. Source data

**Extended Data Fig. 5:**
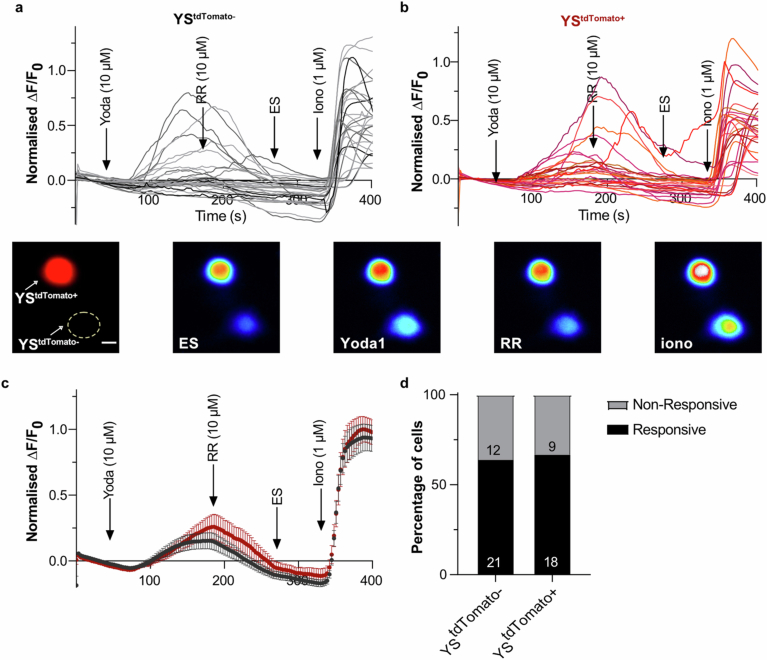
Yolk sac cells respond to Yoda1. **a - b**, *above:* Examples of individual calcium influx responses from YS^tdTomato-^ (grey) and YS^tdTomato+^ (red) cells. The arrows indicate the starting perfusion time of Yoda, Ruthenium red (RR), Extracellular solution (ES) and Ionomycin (iono). *below*: representative images of calcium imaging experiments from YS^tdTomato-^ and YS^tdTomato+^ loaded with Cal520 before (ES) and after perfusion with Yoda1, RR, and Ionomycin. Scale bar 20 µm. **c**, Plot representing the average of the total cells recorded as mean ± S.E.M. **d**, Stacked histograms showing that the proportion of responsive cells to Yoda in YS^tdTomato-^ (63.6%) and YS^tdTomato+^ (66.6%). Numbers indicate number of cells. Six independent experiments were conducted for each group. Source data

**Extended Data Fig. 6:**
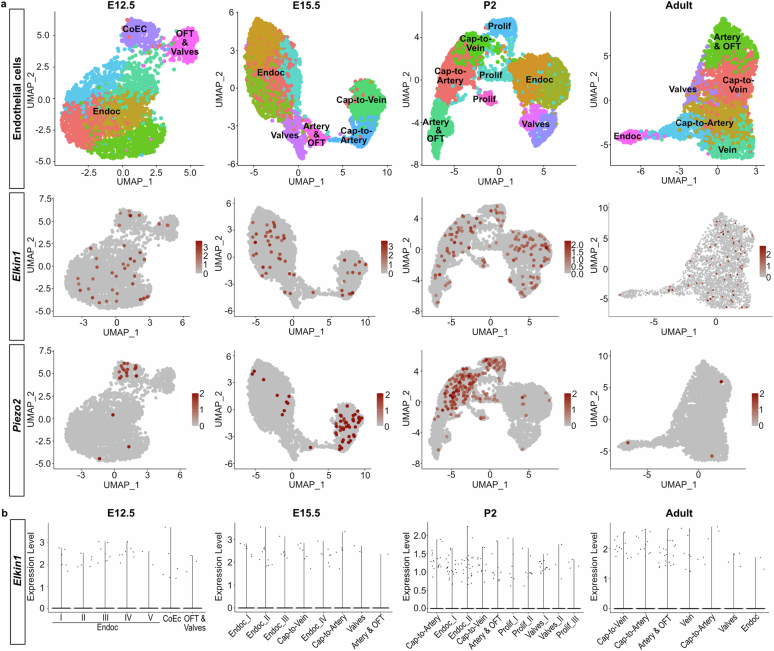
scRNAseq identifies *Elkin1* expression in endothelial cell clusters that are *Piezo2*^*+*^. **a**, UMAP of cardiac endothelial cells from E12.5, E15.5, P2 and murine adult hearts (8 weeks old) showing the following clusters: endocardium (Endoc) that includes five different subclusters_(Endoc_I to Endoc_V), coronary endothelium (CoEC) including the capillary-to-vein (cap-to-vein) and capillary-to-artery (cap-to-artery) clusters, proliferating cells (Prolif_I and Prolif_II), valvular endocardium (Valve_I and Valve_II), arterial and outflow tract cells (Artery & OFT) and venous cells (Vein). **b**, Volcano plots showing *Elkin1* expression levels in cardiac endothelial cells.

The electrophysiological function of PIEZO2 in endothelial cells has not been studied. PIEZO2 ion channels are very efficiently activated by substrate deflection but, unlike PIEZO1 channels, are poorly activated by membrane stretch^31–33^. We identified the yolk sac (YS) from E13.5 to E18.5 embryos as being rich in tdTomato^+^ endothelial cells, which may express functional PIEZO2 channels (Extended Data Fig. 3). We asked whether these cells possess functional mechanosensitive currents characteristic of PIEZO2 channels. We made whole-cell patch–clamp recordings from tdTomato^+^ and tdTomato^−^ YS endothelial cells and cardiac endothelial cells (cECs) cultured on pillar arrays to study currents gated by substrate deflection^31,32^ (Fig. 4a). We also tried cell indentation, a method often used to activate mechanically activated channels^31,32,34,35^, but the endothelial cells were too small and flat to make this method feasible. Nanometer-scale pillar displacements move the substrate–cell contact and are limited to the pillar area (10.3 µm^2^). This mechanical stimulus efficiently evoked inward currents with short latencies (<5 ms) in almost all cells studied owing to the opening of mechanosensitive ion channels sub-adjacent to the stimulated pillus (tdTomato^+^, 12 of 13 cells; tdTomato^−^, 11 of 12 cells). Mechanosensitive currents could be classified into three groups with different inactivation time constants similar to what has been shown in sensory neurons^32^: rapidly adapting (RA) currents (<5 ms), intermediately adapting (IA) currents (5–50 ms) and slowly adapting (SA) currents (>51 ms) (Fig. 4b). Notably, the RA currents possessed ultrafast kinetics in that many of the currents inactivated within a millisecond (Fig. 4c). Very fast inactivation is a characteristic property of PIEZO2 channels, in contrast to PIEZO1 channels that inactivate much more slowly^7,31,32^. Consistently, RA currents with very fast inactivation kinetics were found to be significantly more frequent in tdTomato^+^ cells (chi-squared *P* < 0.05) (Fig. 4b). In addition, mean inactivation time constants for all measured currents were faster in tdTomato^+^ than in tdTomato^−^ cells (Fig. 4c). The frequency with which mechanosensitive currents could be evoked was higher in tdTomato^+^ than in tdTomato^−^ cells, indicating enhanced mechanosensitivity in *Piezo2*^+^ lineage cells. However, no statistically significant differences were observed when comparing deflection–current amplitude relationships (Fig. 4d and Extended Data Fig. 4a). In summary, almost all YS endothelial cells possess very sensitive mechanically activated currents, but cells from the *Piezo2*^*+*^ lineage possessed more currents with kinetics characteristic of recombinantly expressed PIEZO2 channels^7,32^. To directly test what proportion of these mechanically activated currents were dependent on PIEZO2 channels, we used small interfering RNA (siRNA) knockdown targeting *Piezo2* in these cells followed by electrophysiological analysis. We could still measure mechanically activated currents in cells where *Piezo2* was knocked down (Fig. 4e,f and Extended Data Fig. 4d); however, the incidence of such currents was halved compared with controls (Fig. 4g). In control cells, we found mechanically gated currents in all cells measured, but after *Piezo2* knockdown, around a quarter of the cells showed no mechanically gated currents (Extended Data Fig. 4c). The currents measured in the *Piezo2* knockdown cells also tended to have slower inactivation kinetics, which is consistent with a loss of ultrafast inactivating currents mediated by PIEZO2 channels (Fig. 4f). To get closer to the role of mechanosensitive currents in the development of the coronary circulation, we managed to isolate tdTomato^+^ cECs from E13.5 hearts and cultured these cells on pillar arrays. Almost all cells identified as tdTomato^+^ 2 weeks after plating showed very robust and large mechanically activated currents to substrate deflection (14 of 15 cells tdTomato^+^, 7 of 8 cells tdTomato^−^) (Fig. 4a,h–j). However, unlike YS cells, the kinetics and current amplitudes of mechanosensitive currents did not differ between tdTomato^+^ and tdTomato^−^ cells (Fig. 4h,i). We measured absolute deflection thresholds and currents were evoked by movements of less than 50 nm (Extended Data Fig. 4b); a similar sensitivity to that is seen for PIEZO2-dependent touch receptors^32,36,37^. However, the frequency with which mechanosensitive currents could be evoked was higher in cECs that were tdTomato^+^ (Fig. 4j). Thus, there appears to be clear PIEZO2-dependent endothelial cell mechanosensitivity, but other mechanosensitive ion channels such as PIEZO1 and ELKIN1 are probably also present^9^. Consistent with this idea, we could also measure clear stretch-activated currents, typical of PIEZO1 channels, in excised outside-out patches from tdTomato^+^ YS endothelial cells (Extended Data Fig. 4e,f). In addition, calcium influx responses indicated that the PIEZO1 activator Yoda1 (ref. ^38^) induces calcium influx in the majority of tdTomato^+^ and tdTomato^−^ YS cells (Extended Data Fig. 5). Furthermore, 63.6% of the tdTomato^−^ and 66.6% of the tdTomato^+^ YS cells showed a significant YODA1 response, indicating that both PIEZO1 and PIEZO2 are present in YS endothelial cells. Moreover, *Elkin1* transcripts were detected in the same endothelial cell populations in which we had detected *Piezo2* (Extended Data Fig. 6). Thus, apart from PIEZO2, other mechanosensitive ion channels contribute to mechanotransduction in yolk sacs and cECs.

### Pathogenic *Piezo2* mutations cause cardiac hyperplasia

**Fig. 5:**
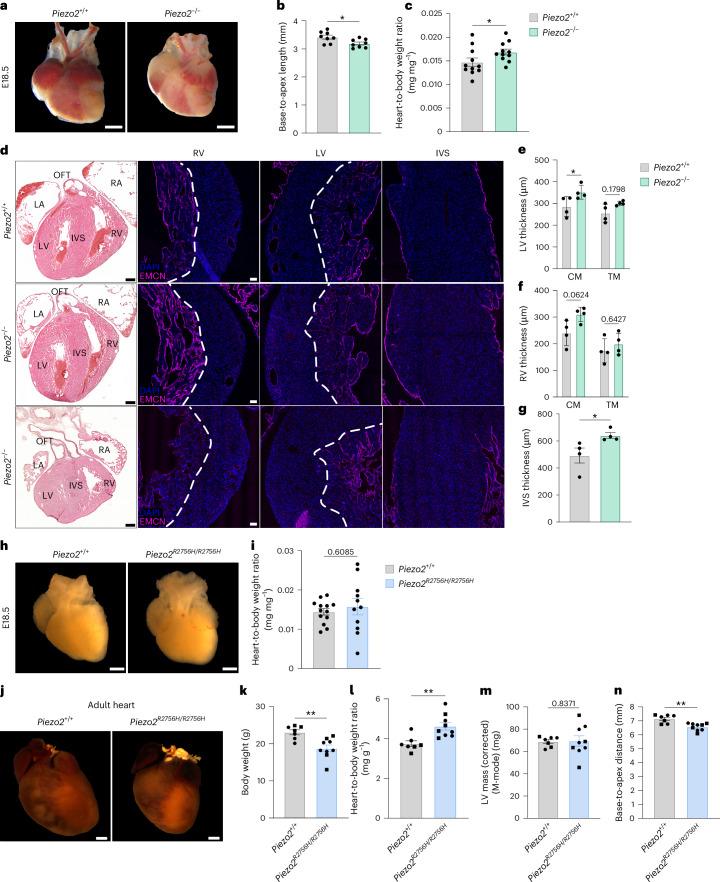
*Piezo2* mutations cause cardiac hyperplasia. **a**, E18.5 *Piezo2*^*+/+*^ and *Piezo2*^*−*^^/^^*−*^ macroscopic analysis. Scale bars, 1 mm. **b**, *Piezo2*^*−*^^/^^*−*^ mutant hearts show a shorter base-to-apex length (*n* = 8 per genotype, Mann–Whitney test, *P* = 0.02). **c**, *Piezo2*^*−*^^/^^*−*^ mutant mice had increased heart-to-body weight ratio (*n* = 11 per genotype, Mann–Whitney test, *P* = 0.04). **d**, Ventricular wall thickness was measured from E18.5 *Piezo2*^+/+^ and *Piezo2*^*−*^^/^^*−*^ hearts immunolabeled against EMCN (magenta) to distinguish between CM and TM, respectively. Dashed lines indicate border between CM and TM. Scale bars, 50 µm. Hematoxylin and eosin staining was performed for an overall morphological heart assessment. Scale bars, 250 µm. Two representative *Piezo2*^*−*^^/^^*−*^ hearts are shown with a milder hyperplasia phenotype (middle row) and a severe hyperplasia phenotype (last row), including the presence of an aberrant outflow tract (OFT). **e**–**g**, *Piezo2*^+/+^ and *Piezo2*^*−*^^/^^*−*^ (*n* = 4 per genotype) LV (**e**), RV (**f**) and IVS (**g**) wall thickness quantification show that E18.5 *Piezo2*^*−*^^/^^*−*^ presents a thicker LV wall and IVS, indicating cardiac hyperplasia (LV, two-way ANOVA, *P* = 0.04 (CM) and *P* = 0.17 (TM); RV, two-way ANOVA, *P* = 0.06 (CM) and *P* = 0.64 (TM); IVS, Mann–Whitney test, two sided, *P* = 0.02). **h**, E18.5 *Piezo2*^*+/+*^ and *Piezo2*^*R2756H/R2756H*^ representative macroscopic images. Scale bars, 1 mm. **i**, *Piezo2*^*R2756H/R2756H*^ E18.5 heart-to-body weight ratio is similar to that of *Piezo2*^*+/+*^ (*n* = 13 *Piezo2*^*+/+*^ and *n* = 11 *Piezo2*^*R2756H/R2756H*^, Mann–Whitney test, *P* = 0.60). **j**, Representative macroscopic heart images of 20-week-old mice carrying a *Piezo2* knock-in gain-of-function mutation (p. Arg2756His) and *Piezo2*^*+/+*^ mice. Scale bars, 1 mm. **k**–**n**, ECG of 10-week-old *Piezo2*^*+/+*^ and *Piezo2*^*R2756H/R2756H*^ mice (*n* = 7 *Piezo2*^*+/+*^, 4 females (dot) and 3 males (square), and *n* = 9 *Piezo2*^*R2756H/R2756H*^, 5 females (dot) and 4 males (square)). **k**, *Piezo2*^*R2756H/R2756H*^ mouse body weight is significantly less than *Piezo2*^+/+^ mouse body weight (Mann–Whitney test, two sided, *P* = 0.003). **l**, The heart-to-body weight ratio is increased in *Piezo2*^*R2756H/R2756H*^ mutants compared with *Piezo2*^+/+^ (Mann–Whitney test, two sided, *P* = 0.003). **m**, Both groups had similar LV mass (Mann–Whitney test, two sided, *P* = 0.83). **n**, Heart size was measured from base to apex showing that *Piezo2*^*R2756H/R2756H*^ hearts are smaller than *Piezo2*^*+/+*^ hearts (Mann–Whitney test, two sided, *P* = 0.004), indicating hyperplasia or hypertrophy. *P < 0.05; **P < 0.01. All error bars represent ±s.e.m. Source data

**Extended Data Fig. 7:**
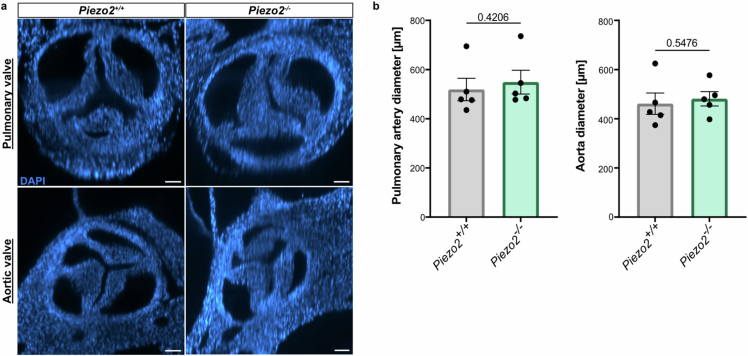
*Piezo2* mutations do not alter the cardiac valve and OFT morphology. **a**, Representative images of the aortic and pulmonary valves of *Piezo2*^*+/+*^ and *Piezo2*^*-/-*^ E18.5 embryos. Optical sections from whole mount light sheet images are shown. Nuclei are stained with DAPI. **b**, *Piezo2*^-/-^ mutants present a similar pulmonary artery and aorta diameter compared to Piezo2^+/+^ (n = 5 per genotype, Mann-Whitney test, two-sided, *P* = 0.420 [Pulmonary artery] and *P* = 0.547 [Aorta]. Scale bars: 100 µm). Error bars represent ± S.E.M. Source data

We next asked whether PIEZO2 channels are necessary for normal heart development. As *Piezo2*^*−/*^^*−*^ mutant mice die perinatally^14,15^, we examined hearts from these mice at E18.5. *Piezo2*^*−*^^/^^*−*^ mutant hearts were smaller and shorter (base to apex length) at birth than those of wild-type (WT) *Piezo2*^*+/+*^ littermates (Fig. 5a,b). However, measuring heart-to-body weight ratios, we found that the ratios were increased in mutants, indicating that *Piezo2*^*−*^^/^^*−*^ embryonic hearts were heavier than WT hearts (Fig. 5c). We made a detailed morphological analysis using the endocardial marker EMCN to differentiate between the trabecular myocardium (TM) and compact myocardium (CM) (Fig. 5d). The results revealed that *Piezo2*^*−*^^/^^*−*^ hearts showed clear ventricular hyperplasia, which was most evident for the left ventricle (LV) (Fig. 5e,f). In addition, the interventricular septum (IVS) was also substantially thickened (Fig. 5g). No obvious outflow tract defects, such as common arterial trunk and double outflow tract right ventricle (RV), were observed in the *Piezo2*^*−*^^/^^*−*^ mutants. Pulmonary artery and ascending aorta diameters were comparable between WT and *Piezo2*^*−*^^/^^*−*^ hearts (Extended Data Fig. 7). Longitudinal and transverse sections of the valves showed clear separation of the leaflets (Extended Data Fig. 7).

Rare *PIEZO2* pathogenic variants in humans are associated with neurodevelopmental disorders such as Gordon syndrome and Marden–Walker syndrome as well as distal arthrogryposis^16,17^. We generated mice carrying a pathogenic homozygous gain-of-function mutation (*Piezo2*^*R2756H/R2756H*^) that dramatically sensitizes the activation of endogenous PIEZO2 channels by relieving voltage block of the channel^39^. At E18.5, *Piezo2*^*R2756H/R2756H*^ mutant hearts showed no overt phenotype with similar size (Fig. 5h) and heart-to-body weight ratio compared with WT littermate controls (Fig. 5i). As homozygous *Piezo2*^*R2756H/R2756H*^ mutants are viable, we examined the hearts of these animals at 20 weeks of age postmortem (Fig. 5j). Similarly to embryonic *Piezo2*^−/−^ hearts, we found that the *Piezo2*^*R2756H/R2756H*^ mutant hearts were smaller and shorter than hearts from age-matched WT littermates. We noted that *Piezo2*^*R2756H/R2756H*^ mutant mice at 10 weeks weighed significantly less than WT littermates (Fig. 5k). Indeed, echocardiography (ECG) measurements from living *Piezo2*^*R2756H/R2756H*^ and WT littermate mice at 10 weeks of age showed that *Piezo2*^*R2756H/R2756H*^ mutant mice displayed a significantly elevated heart-to-body weight ratio compared with controls (Fig. 5l). LV mass was comparable between genotypes (Fig. 5m), but mutant hearts were significantly shorter than WT hearts (Fig. 5n). These results suggest that both loss- and gain-of-function PIEZO2 can lead to macroscopic hyperplastic changes in the embryonic and adult heart.

### PIEZO2 shapes normal coronary artery development

**Fig. 6:**
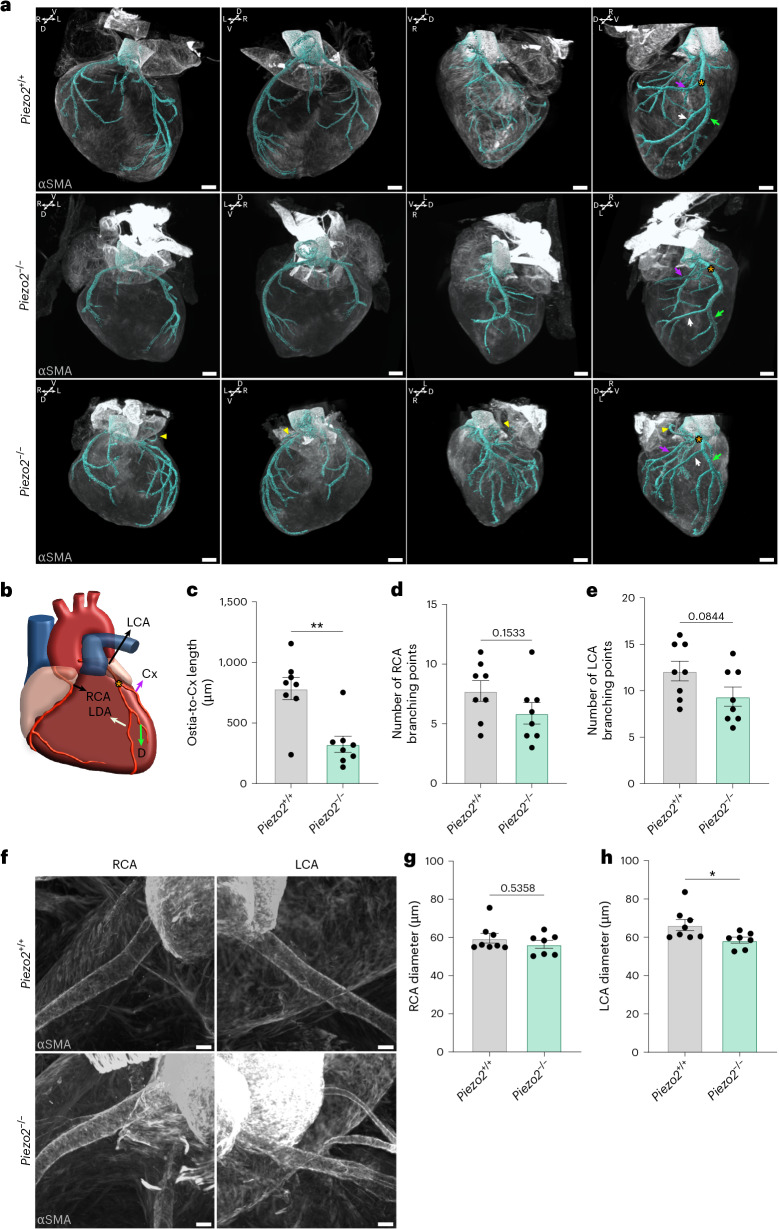
*Piezo2* loss-of-function mutations alter coronary artery development. **a**, A 3D rendering of representative *Piezo2*^*+/+*^ and *Piezo2*^*−*^^/^^*−*^ E18.5 hearts immunolabeled against αSMA (gray), a smooth muscle marker, used for coronary vasculature segmentation (cyan). Scale bars, 300 µm. **b**, The schematic, generated in affinity designer 2, shows the RCA and LCA and the LCA main branches: the Cx (magenta arrow), D (green arrow) and the LDA (white arrow). The orange asterisk depicts the Cx bifurcation point. The yellow arrowheads on the mutant heart point to an aberrant ectopic coronary branch in a *Piezo2*^*−*^^/^^*−*^ heart. **c**, The circumflex artery of *Piezo2*^*−*^^/^^*−*^ hearts branches closer to the left coronary artery ostia (*n* = 8 per genotype, Mann–Whitney test, *P* = 0.007). **d**,**e**, Number of RCA (**d**) and LCA (**e**) branching points (unpaired Student’s *t*-test, *P* = 0.153 (RCA) and *P* = 0.08 (LCA)). **f**–**h**, RCA (**g**) and LCA (**h**) diameters were measured and compared between *Piezo2*^*+/+*^ (*n* = 8) and *Piezo2*^*−*^^/^^*−*^ (*n* = 7). *Piezo2*^*−*^^/^^*−*^ presents with a significantly smaller left LCA diameter (Mann–Whitney test, *P* = 0.02 (LCA in **h**) and *P* = 0.53 (RCA in **g**)). Representative images are shown in **f**. Scale bars, 50 µm. *P < 0.05; **P < 0.01. All tests were two sided and error bars represent ±s.e.m. Source data

**Extended Data Fig. 8:**
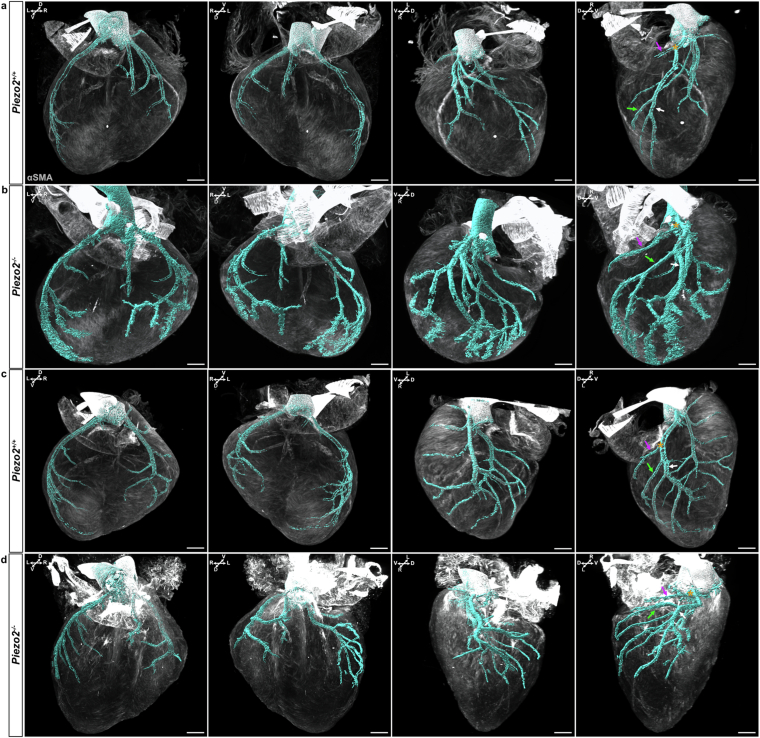
Three-dimensional rendering of *Piezo2*^*+/+*^ and *Piezo2*^*-/-*^ E18.5 hearts. **a - d**, iDisco-cleared and αSMA (gray) immunolabelled hearts from *Piezo2*^*+/+*^ wild types (**a** and **c**) and *Piezo2*^*-/-*^ mutants (**b** and **d**), used for coronary vasculature reconstruction (cyan) (Scale bars: 300 µm). Arrows indicate the left coronary artery branches: the circumflex artery (magenta arrow), the diagonal branch (green arrow) and the left descending artery (white arrow). The orange asterisk shows the branching point of the circumflex artery.

The LV hyperplasia observed in *Piezo2*^*−*^^/^^*−*^ mice is a phenomenon that can result from reduced perfusion of the cardiac muscle. As PIEZO2 is highly specific to the coronary endothelium, we hypothesized that PIEZO2 mechanosensory function in these cells guides the normal assembly of the coronary vasculature. We performed whole-organ immunostaining against αSMA in immunolabeling-enabled three-dimensional imaging of solvent-cleared organs (iDISCO)-cleared embryonic hearts. Using light-sheet imaging and vessel segmentation, we could visualize the entire coronary vasculature in WT and *Piezo2*^*−*^^/^^*−*^ hearts (Fig. 6a,b, Extended Data Fig. 8 and Supplementary Videos 3–18). The majority of the main branches of the coronary artery network follow a stereotypical pattern^40^. From the coronary artery ostium at the left coronary cusp of the ascending aorta, the left main coronary artery (LCA) extends ventrally before the first bifurcation into the left anterior descending artery (LDA) and left circumflex artery (Cx). Further ventrally, the diagonal branch (D) bifurcates from the LDA. Conversely, the right coronary artery (RCA) connects to the aorta at the right coronary cusp.

**Extended Data Fig. 9:**
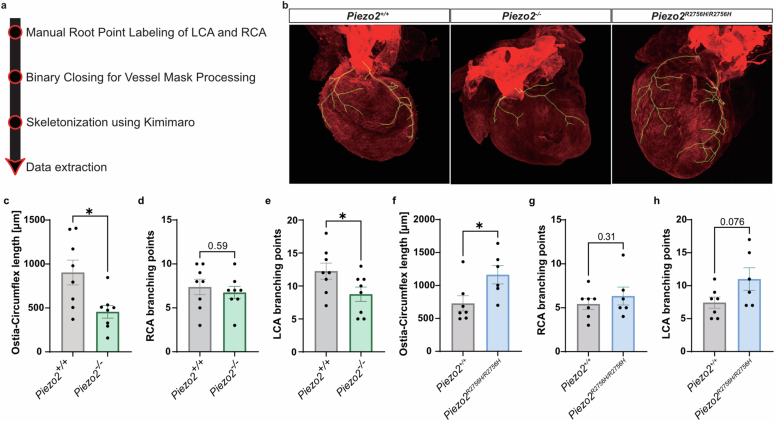
Computer-vision based 3D artery reco nstruction for unbiased artery parameter measurement. **a**, A simple workflow for Computer-vison based 3D artery reconstruction (see methods). **b**, three representative images with artery centerline generated by this method, for *Piezo2*^*+/+*^*, Piezo2*^*-/-*^ and *Piezo2*^*R2756H/R2756H*^ hearts, respectively at E18.5. **c - e**, Same parameters measures as in Fig. 6c–e. (Unpaired Student t-test, two-sided, *P* = 0.014 [ostia to circumflex length in panel **c**], *P* = 0.59 [RCA branching point number in panel **d**], *P* = 0.049 [LCA branching point number in panel **e**]). **f - h**, Same parameters measures as in Fig. 7b–d (Unpaired Student t-test, two-sided, *P* = 0.034 [ostia to circumflex length in panel **f**], *P* = 0.31 [RCA branching point number in panel **g**], *P* = 0.076 [LCA branching point number in panel **h**]). * *P* < 0.05. Error bars represent ± S.E.M. Source data

In *Piezo2*^*−*^^/^^*−*^ hearts, the coronary vasculature topology was clearly altered compared with the WT. In particular, the circumflex artery branched off from the left coronary artery closer to the ostia (Fig. 6a (orange asterisks) and Extended Data Fig. 8 (orange asterisks)). Quantitative analyses revealed a significantly shorter segment length between the coronary ostia and the Cx branching point in *Piezo2*^*−*^^/^^*−*^ hearts compared with controls (Fig. 6c and Extended Data Fig. 9c). Moreover, in *Piezo2*^*−*^^/^^*−*^ hearts, the branching of the left coronary artery into the Cx, D and the LDA did not follow the pattern seen in the WT. There was no clear distinction between the main LDA and its branches in the mutant heart compared with the WT (Fig. 6a (white arrows) and Extended Data Fig. 8 (white arrows)). The branch points in mutant vessels were frequently trifurcations rather than bifurcations. In addition, in one mutant heart, an ectopic artery branching off the Cx projecting toward the anterior pole of the heart was observed (Fig. 6a (yellow arrowheads)). Manual and computer-vision-based quantification of branch points revealed that while the number of branch points was not altered in the RCA tree of *Piezo2*^*−*^^/^^*−*^ mutants (Fig. 6d and Extended Data Fig. 9d), fewer branch points were observed in the left coronary artery tree of mutants compared with controls (Fig. 6e and Extended Data Fig. 9e).

Most importantly, the light-sheet reconstruction indicated that *Piezo2*^*−*^^/^^*−*^ hearts have more constricted coronary vessels. As LV hyperplasia was most prominent in *Piezo2*^*−*^^/^^*−*^ hearts, we quantified the average diameter of the left and right main coronary artery over 600 µm from the ostia (Fig. 6f–h). We observed significant narrowing of the left coronary artery diameter in *Piezo2*^*−*^^/^^*−*^ hearts compared with the WT (Fig. 6h).

To assess potential perfusion changes caused by the LCA constriction, we applied physical first principles. We considered a cylindrical pipe of constant cross-section where an incompressible, Newtonian fluid is flowing under laminar flow conditions as an idealized model of the LCA. Poiseuille’s law relates the flow rate in the pipe *Q*, the pressure drop between both ends of the pipe Δ*P* and the resistance of the pipe *R* (equation (1))

1 $$\Delta P={RQ}$$

2 $$R=\frac{8\mu L}{\pi {r}^{4}}$$

where *μ* is the constant dynamic viscosity of the fluid, *L* is the length of the pipe and *r* is the radius of the pipe (equation (2))^41^. The LCA diameter has narrowed, on average, from 66.377 μm to 58.492 μm, that is, an 11.879% reduction in *Piezo2*^*−*^^/^^*−*^ hearts compared with *Piezo2*^+/+^ controls. Given a comparable pressure drop between the entrance of the LCA and its post-capillary venous return in both the *Piezo2*^+/+^ and *Piezo2*^*−*^^/^^*−*^ groups (as can be expected from a hierarchically branched vascular tree with multiple major arteries running in parallel, for example, LCA and RCA), the fact that the largest pressure drop along this path happens in small arteries and arterioles^42^, and all the remaining variables being equal, it follows from the previous equations and assumptions that the resistance of the LCA will have increased by 65.7% in *Piezo2*^*−*^^/^^*−*^ hearts and that this will lead to a very significant reduction of perfusion downstream from it despite the relatively modest reduction in LCA diameter.

**Fig. 7:**
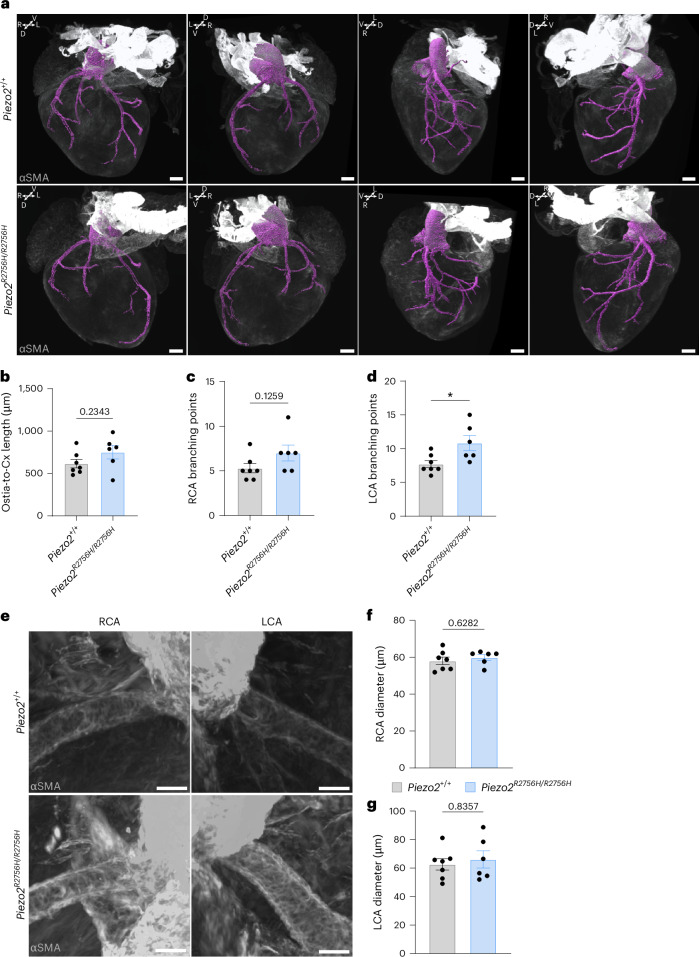
*Piezo2* gain-of-function mutations alter coronary artery development. **a**, A 3D rendering of representative *Piezo2*^*+/+*^ and *Piezo2*^*R2756H/2756H*^ E18.5 hearts immunolabeled against αSMA (gray), a smooth muscle marker, used for coronary vasculature segmentation (cyan). Scale bars, 300 µm. **b**–**d**, Analyses of the coronary vascular tree topology, showing that *Piezo2*^*R2756H/R2756H*^ presents a significantly increased number of LCA branching points (BP) (*n* = 7 *Piezo2*^*+/+*^ and *n* = 6 *Piezo2*^*R2756H/R2756H*^, Mann–Whitney test, *P* = 0.234 (ostia-to-circumflex length in **b**), *P* = 0.125 (RCA BP in **c**) and *P* = 0.023 (LCA BP in **d**)). **e**–**g**, RCA and LCA diameters were measured and compared between *Piezo2*^*+/+*^ (*n* = 7) and *Piezo2*^*R2756H/R2756H*^ (*n* = 6) (Mann–Whitney test, *P* = 0.628 (RCA in **f**) and *P* = 0.835 (LCA in **g**)). Representative images are shown in **e**. Scale bars, 50 µm. *P < 0.05. All tests were two sided and error bars represent ±s.e.m. Source data

We next assessed the coronary artery topology in E18.5 *Piezo2*^*R2756H/R2756H*^ hearts using iDISCO clearing, αSMA immunostaining and light-sheet imaging followed by vessel segmentation and coronary vasculature surface reconstruction in a total of six *Piezo2*^*R2756H/R2756H*^ mutant and seven WT control samples (Fig. 7a and Supplementary Videos 19–31). Quantification of segment length and branch point numbers was conducted by two different methods (manual and computer vision based) and performed by different researchers in a blinded manner.

Quantitative assessments of the coronary artery topology revealed that the length of the segment between the coronary ostia and the Cx branching point was slightly increased in *Piezo2*^*R2756H/R2756H*^ mutant hearts at E18.5 compared with controls (Fig. 7b and Extended Data Fig. 9f). Moreover, the number of total left coronary artery branch points was increased in *Piezo2*^*R2756H/R2756H*^ gain-of-function mutant hearts (Fig. 7d and Extended Data Fig. 9h).

Taking these results together, we conclude that the proper function of the mechanosensitive PIEZO2 ion channels is necessary for the normal assembly of the cardiac endothelium to form an adequate vasculature supply to the developing myocardium.

## Discussion

Our investigation reveals a critical role of mechanosensitive PIEZO2 function in the formation of the coronary vasculature, particularly in developing coronary endothelial cells. We detected a transient expression for *Piezo2* within the *Fabp4*^+^ endothelial cell population in the heart, which peaked during the embryonic and early postnatal stages but diminished in adulthood, suggesting that PIEZO2 is crucial for coronary vessel formation but not for maintaining mature endothelial identity under healthy conditions. *Piezo2-*expressing coronary endothelial cells show unique expression profiles characterized by the enrichment of genes involved in cell migration, guidance and angiogenesis, including *neuropilin 2*, *apelin* and *Crmp1* (ref. ^23^). Neuropilin 2, originally recognized for its role in neuronal guidance^25^, also influences angiogenesis and probably guides endothelial cell migration and vessel formation^23^. Similarly, apelin, a hormone known for supporting angiogenesis, enhances endothelial cell survival and stabilizes blood vessels^43^. In addition, CRMP isoforms, initially associated with neuronal development^26^, are present in endothelial cells, where they probably contribute to cytoskeletal regulation, cell movement and angiogenesis.

Of note, *PIEZO2* expression has been reported in human cultured coronary artery endothelial cells^44^ and in endothelial cells of cardiac tissue samples from transplanted human hearts^45^. In an scRNA-seq analysis of P7 murine brain endothelial cells, it was shown that *Piezo2* was among the 25 most enriched tip cell transcripts^46^. PIEZO2 has been implicated in transducing physical cues into mechanobiological responses, such as cytoskeletal rearrangements^47^. It is therefore plausible to hypothesize that PIEZO2 is needed to guide endothelial navigation at the angiogenic front of the coronary vascular plexus in response to mechanical cues. Additional previously published data further support a potential role for PIEZO2 in endothelial cell motility. Yang et al. described that *PIEZO2* siRNA-treated HUVEC cells showed a significant decrease in tube formation compared with the control group. They also showed that shRNA-mediated *Piezo2* knockdown in mice caused a significant reduction in the radial extension of vascular plexus from the optic nerve to the periphery at P3 and P5, as well as fewer branch points^12^. Wei et al. describe impaired cell migration and tube formation in pulmonary microvasculature endothelial cells isolated from endothelial cell-specific *Piezo2*^EC/−^ knockout rats compared with WT rats^13^.

Chemical signaling (chemotaxis), particularly through pathways such as VEGFA-VEGFR2, BMP2 and CXCL12/CXCR4, is well established in orchestrating endothelial cell movements underlying angiogenesis in various organs, including the heart^3,4^. However, there is increasing evidence that combinatorial effects of chemotaxis and ion channel-mediated durotaxis fine-tune cellular migration and navigation in diverse cellular systems^5,48,49^. In particular, the study by Canales Coutiño and Mayor in *Xenopus* showed that Piezo1 cooperates with chemotactic signaling cues to control neural crest migration^48^. PIEZO2 was also shown to balance mechanosensitivity and chemosensitivity at the blood–tumor barrier in medulloblastoma^50^.

Indeed, endothelial PIEZO2 could be a major sensor of mechanical cues in the developing myocardium, facilitating cellular migration and motility through durotaxis. We propose that mechanical cues sensed by *Piezo2*^*+*^ coronary endothelial cells are crucial for precise cellular navigation during coronary vasculature plexus formation and remodeling.

Our functional analysis showed that *Piezo2*^+^ endothelial cells possess fast mechanosensitive currents that have sensitivities and kinetics similar to those of *Piezo2*-dependent currents in sensory cells^32,36,39^. However, many cECs that had never expressed *Piezo2* also exhibited mechanosensitive currents (Fig. 4), showing that PIEZO2 is only one of several functional mechanosensitive channels in cECs^51^. Using a knockdown approach in YS cells, we show directly that PIEZO2 contributes to mechanically gated currents in these cells, but it is clearly not the sole mechanosensitive ion channel present (Fig. 4). Indeed, our data show that *Elkin1* and *Piezo1* are also expressed in cECs, and both channels are efficiently gated by substrate deflection or membrane stretch^32^. Nevertheless, it is striking that PIEZO2 loss- and gain-of-function experiments revealed a nonredundant role of endothelial PIEZO2-dependent mechanosensitivity in shaping coronary artery formation and branching.

In the absence of *Piezo2* expression, we observed aberrant coronary artery branching, that is, significantly decreased proximal left coronary artery segment length and less branch points of the left coronary artery tree and most importantly a decreased left coronary artery diameter. We propose that cardiac muscle perfusion deficits result from coronary anomalies in *Piezo2*^−^^/^^−^ mutants during embryonic development. Reduced blood supply could lead to hypoxia-induced compensatory growth. Hemodynamic stress could additionally be a cause of heart malformations and hyperplastic myocardial growth observed in PIEZO2-deficient mouse embryos (Fig. 5a–g). Human pathogenic gain-of-function mutations in *PIEZO2*, documented in human patients^16,17^, dramatically increase the channels’ sensitivity to mechanical force^39^. By introducing this gain-of-function mutation into mice, we show here that PIEZO2 hypersensitivity can also lead to coronary artery branch point anomalies (Fig. 7d and Extended Data Fig. 9f–h) and disruption of postnatal heart development (Fig. 5j–n). The increased number of branch points in the left coronary artery of *Piezo2*^*R2756H/R2756H*^ gain-of-function mutants, even with a normal proximal LCA diameter, could potentially lead to cardiac hyperplasia and hypertrophy. Increased local shear stress due to more branch points and increased local perfusion could stimulate cell cardiomyocyte proliferation in the embryo or cell growth in the adult.

However, a direct functional link between PIEZO2 dysfunction in coronary arteries and cardiac muscle anomalies still needs further experimental evidence and a potential role of PIEZO2 in the outflow tract and the valves of cardiovascular phenotypes needs to be assessed in further studies.

While the main causes of coronary artery disease are adult-onset conditions such as atherosclerosis, hypertension and diabetes due to lifestyle modifications, genetic predisposition can also have an important role in disease etiology^52^. Congenital coronary artery defects have been implicated in cardiac hyperplasia and hypertrophy^53^, and although they might be mild without any symptoms during development and in adolescence, they are considered important factors that can predispose individuals to cardiac disease in adult life^54^. Indeed, the heart malformations that we have observed are likely to contribute to early perinatal mortality in *Piezo2*^−^^/^^−^ mice, which was attributed to failure to inflate the lungs after birth^14,15^.

The importance of PIEZO2 in a variety of sensory functions, including pain, makes it a potentially attractive target for drug therapies. However, our findings indicate that PIEZO2 is also crucial for the development of coronary arteries in the heart. Considering that developmental programs are often reused in regeneration and remodeling conditions, PIEZO2 might be a promising candidate to be reexpressed in the adult heart to facilitate angiogenesis following cardiac ischemic episodes. Findings from recent studies support this hypothesis, for example, in rodents, Kloth and colleagues observed a clear upregulation of *Piezo2* in stressed cardiac tissue^55^. Further studies describe upregulation of Piezo2 in pharmacologically stressed cultured human coronary artery endothelial cells^44^ and in cardiac samples from patients with heart failure^56^. Indeed, cardiovascular problems have been associated with *PIEZO2* pathogenic variants in a small number of human patients^57,58^ and the University of Cambridge PhenoScanner V2 database of human genotype–phenotype associations suggests *PIEZO2* genetic linkage with heart failure, diastolic blood pressure, hypertensive heart disease and thoracic aortic aneurysm^18^.

In summary, our study elucidates the indispensable role of PIEZO2-dependent mechanosensitive signaling in coronary vasculature formation and highlights its implications for cardiac development and disease. Our study also raises a new issue for the use of therapeutics targeting PIEZO2 in the somatosensory system as such approaches may have serious consequences for cardiovascular (patho)physiology.

## Methods

### Animal care

All mouse experiments complied with the German Animal Protection Act and were approved by the Max Delbrück Center for Molecular Medicine and the local Berlin authority, Landesamt für Gesundheit und Soziales Berlin, under the following animal protocol numbers: G0233/19, G0017/17 and X9006/21. Mice were housed under standard conditions (22 ± 2 °C, 55 ± 10% humidity, 12:12 h light–dark cycle) with ad libitum access to food and water, and provided with nesting and hiding materials. Timed matings using mice >10 weeks old were set up to collect embryos at specific stages (plug day = E0.5). Pregnant females were killed by cervical dislocation at the desired embryonic stage.

### Genetically modified mice

B6.Cg-Gt*(ROSA)26Sor*^*tm14(CAG-tdTomato)Hze/J*^: Ai14 is a Cre reporter strain (The Jackson Laboratory, 007908) and expresses tdTomato fluorescence following Cre-mediated recombination. Ai14 mice were used for timed matings with B6(SJL)-*Piezo2*^*tm1.1(cre)Apat/J*^ mice (The Jackson Laboratory, 027719)^15^, herein referred to as Piezo2-Cre mice, to obtain Piezo2-cre; Ai14 embryos expressing dtTomato in the *Piezo2*^*+*^ lineage (herein referred to as *Piezo2*-tdTomato mice).

Generation of the *Piezo2* null allele (Piezo2^−^) was originally achieved by crossing the B6(SJL)-*Piezo2*^*tm2.2Apat/J*^ strain (The Jackson Laboratory, 027720) with the E2a-cre strain (FVB/N-Tg(EIIa-cre)C5379Lmgd/J; The Jackson Laboratory, 003314) as described in Extended Data Fig. 3 from Woo et al.^15^. EIIa-cre-mediated recombination leads to germline transmission of the recombined Piezo2 allele. *Piezo2*^*−*^^/^^*−*^ embryos were isolated after timed matings of *Piezo2*^+/^^*−*^ parents >10 weeks.

The *Piezo2*^*R2756H/R2756H*^ strain was generated as described in Sánchez-Carranza et al.^39^. All these strains were kept on a C57BL/6N background (backcross onto C57BL/6N from C57BL/6J for more than 12 generations).

### Immunohistochemistry

Mouse embryonic hearts were fixed in 4% paraformaldehyde in phosphate-buffered saline (PBS) for 2 h at room temperature (RT), washed in 1× PBS, cryopreserved in 30% sucrose, embedded in optimal cutting temperature embedding matrix (Tissue-Tek, Sakura Finetek) and sectioned at 10 µm using a Leica CM1950 cryostat. Sections were permeabilized with 0.1% Triton X-100 for 30 min and blocked with 10% donkey serum (Biowest) and 1% bovine serum albumin (BSA; Sigma-Aldrich) for 1 h at RT. Primary antibodies diluted in phosphate-buffered saline with Tween 20 (PBST) with 1% serum were applied overnight at 4 °C, followed by secondary antibodies for 1 h at RT. 4′,6-Diamidino-2-phenylindole (DAPI; 62248, Invitrogen) was used for nuclear counterstaining, and DAKO fluorescent medium (S302380-2, Agilent) was used for mounting.

Whole-mount immunostaining of E13.5 hearts was performed in 2-ml Eppendorf tubes under rotation. Hearts were permeabilized with 0.5% Triton X-100 in PBS for 1 h, then blocked with 10% donkey serum in 0.5% PBST for 2 h at RT. Primary and secondary antibodies were applied sequentially for 24 h at 4 °C, with PBST washes between steps. DAPI was used for nuclear counterstaining. Samples were mounted in µ-slide eight-well glass-bottom chambers (IBIDI) using Fluoromount G (00-4959-52, eBioscience).

### Tissue clearing and immunolabeling

E18.5 Piezo2-tdTomato hearts were cleared using the SmartClear protocol (LifeCanvas Technologies), which preserves endogenous fluorescence. Before clearing, paraformaldehyde-fixed samples underwent SHIELD epoxy-based fixation. Clearing was performed for 3 days at 42 °C using the SmartClear device. Samples were incubated overnight at 37 °C in 50% EasyIndex and distilled water with shaking, then in 100% EasyIndex for refractive index matching.

E11.5 Piezo2-tdTomato and E18.5 *Piezo2*^*−*^^/^^*−*^ hearts were cleared and labeled using an optimized iDISCO protocol^59^. Samples were dehydrated, washed and incubated in 66% dichloromethane–33% methanol overnight at RT, then bleached overnight in 5% H_2_O_2_–methanol, rehydrated, washed and permeabilized. Samples were blocked and incubated in antibody solution for 4 days at 37 °C. Hearts were embedded in 1% low-melting agarose, dehydrated, incubated for 2.5 h in 66% dichloromethane–33% methanol, followed by 100% dichloromethane, and stored at RT in ethyl cinnamate.

### Antibodies

The following antibodies were used: goat anti-PECAM1 (1:250, AF3628, R&D Systems), rabbit anti-DACH1 (1:100, 10910-1-AP, Proteintech), rat anti-EMCN (1:100, clone V.C7C7, sc-65495, Santa Cruz), rabbit anti-FABP4 (1:100, ab13979, Abcam), goat anti-SOX17 (1:100, AF1924, R&D Systems), rabbit anti-RFP (1:1000, 600-401-3979S, Rockland), rat anti-VE-Cadherin (555289, clone 11D4.1, BD-Pharmigen), mouse anti-SMA (1:200, clone 11D4.1, A5228, Sigma-Aldrich), mouse anti-SMA-Cy3 (1:250, clone 1A4, C6198, Sigma-Aldrich), Alexa Fluor 488 rat (1:500, ab150153, Abcam), Alexa Fluor 555 rabbit (1:500, ab150074, Abcam), Alexa Fluor 647 rabbit (1:500, ab150075, Abcam), Alexa Fluor 647 goat (1:500, ab150131, Abcam) and Alexa Fluor 647 rat (1:500, ab150155, Abcam).

### Hematoxylin and eosin staining

Hematoxylin and eosin staining was performed, following the manufacturer’s instructions on 10-µm-thick cryosections. Stained sections were imaged using a Leica DM5000B microscope.

### Single-molecule fluorescent in situ hybridization

Single-molecule fluorescent in situ hybridization was carried out according to the manufacturer’s instructions (RNAscope Multiplex Fluorescent V2 assay, 323110, ACD) on E13.5 WT heart sections. *Piezo2* (Probe-Mm-*Piezo2*, 400191, ACD) and *Dach1* (Probe-Mm-*Dach1*, 12071-C3, ACD) expression was studied. Opal 570 (FP1488001KT, Akoya Biosciences) and Opal 690 (FP1497001KT, Akoya Biosciences) fluorophores were used.

### Confocal imaging

Immunofluorescently stained tissue sections and whole-mount E11.5 and E13.5 hearts were imaged using an LSM700 confocal microscope with ZEN software. Imaging was performed using ×10 NA 0.3 EC Plan-Neofluar, ×20 NA 0.8 Plan Apochromat and ×40 NA 1.4 Oil Plan Apochromat objectives. Fluorophores were excited and detected as follows: DAPI (405 nm excitation, 420–450 nm detection), Alexa Fluor 488 (488 nm, 500–550 nm), Alexa Fluor 555 (555 nm, 570–620 nm) and Alexa Fluor 647 (639 nm, 660–730 nm). The pinhole was set to 1 AU for all channels.

### Light-sheet microscopy

Cleared and immunolabeled whole E18.5 hearts were imaged with a Zeiss Lightsheet 7 microscope, with the ZEN 3.1 (black edition) LS software. The acquisition was done with dual-side illumination (6.08 µm light-sheet thickness), using LSFM ×5/0.1 foc objectives for illumination and an EC Plan-Neofluar ×5/0.16 foc lens for detection, with the correction collars adjusted to the correct refractive index, depending on the immersion used (1.46 for EasyIndex, 1.56 for Ethylene Cinnamate). Acquisition was done sequentially, using solid-state lasers 405 nm and 561 nm (camera beamsplitter LP 510 nm, emission filters BP (420–470) nm/(575–615) nm), with a PCO.Edge 5.5 sCMOS camera (6.5 µm per pixel, 1,920 × 1,920 pixels), and using a zoom of 0.96 (0.97 × 0.97 × 2.00 µm per voxel). Post-acquisition processing was performed with the ZEN 3.4 (blue edition) software to fuse the dual-side light sheets of each sample while the FIJI BigStitcher plugin was used to stitch the tiles.

### Image analysis

Identical settings for laser power, detector and pixel size were used for all samples analyzed qualitatively or quantitatively. For image post-processing (FIJI 2.14.0), all parameters were uniformly applied across all samples.

Ventricular wall thickness was measured from longitudinal sections of E18.5 *Piezo2*^+/+^ and *Piezo2*^−^^/^^−^ E18.5 hearts. Six sections per animal were selected, three with the pulmonary artery and three with the aorta. Measurements were taken, using FIJI, from the LV, the RV and the IVS. A region of interest below the atria was defined to measure compact and TM thickness. Ten measurements were taken per area per section. The average thickness per area per animal was used for final quantification.

Right and left coronary artery diameters were measured in FIJI from whole-mount E18.5 *Piezo2*^+/+^ and *Piezo2*^−^^/^^−^ immunolabeled hearts. Measurements were taken precisely at 200 µm, 300 µm, 400 µm, 500 µm and 600 µm from the connection of the artery to the aorta. These five values per artery were averaged and used in statistical analysis.

The diameters of the pulmonary artery and the aorta were measured using FIJI from optical sections obtained from whole-mount E18.5 *Piezo2*^+/+^ and *Piezo2*^−^^/^^−^ immunolabeled hearts. Two measurements were taken per vessel and averaged. The resulting values were used for the statistical analysis.

Coronary vasculature surface reconstruction was based on the iDISCO-cleared, αSMA-immunolabeled *Piezo2*^*+/+*^ and *Piezo2*^−^^/^^−^ hearts. The surface package of IMARIS 10.0 was used to semi-automatically detect, segment and reconstruct the immunofluorescence signal. Segmentation and 3D reconstruction were used for qualitative and quantitative coronary architecture analyses. Filaments plugin from IMARIS was used to measure the ostia-to-circumflex artery length.

### Computer-vision-based 3D coronary artery reconstruction

Branching point analysis was based on the surface reconstruction of the coronary vasculature. We manually labeled the coronary artery root points for the right and left ventricles and refined segmentation masks using napari 0.5.6 (https://napari.org/stable/). Unwanted regions outside the right and left ventricles were removed and broken structures reconnected. Binary closing was used to fill the interior of the blood vessels and skeletonized the resulting 3D masks using Kimimaro (https://github.com/seung-lab/kimimaro), with roots as starting points. From these skeletons, we computed the distances to each branching point and the total number of branching points. We manually identified the branching points of interest and recorded their measurements (ostia–circumflex). The code is available at https://github.com/Kainmueller-Lab/piezo2_branching_point_analysis.

The code has been deposited in a capsule with Code Ocean.

### ECG

Ultrasound examination was performed on anesthetized 10-week-old *Piezo2*^*+/+*^ and *Piezo2*^*R2756H/R2756H*^ mice using the Vevo 3100 system (VisualSonics Fujifilm) with an MXD550D ultrasonic probe. Anesthesia was induced with 3–4% isoflurane (anesthesia box) and maintained with 1.5–2% isoflurane (mask ventilation). The examination area was shaved and treated with hair removal cream; eye ointment (Bepanthen) was applied to prevent dryness. The mice were placed on a warming plate with additional heat from a lamp, and body temperature was monitored via a rectal probe. ECG was recorded throughout to monitor heart rate and anesthesia depth. Contact gel ensured proper coupling between the transducer and skin. The examinations lasted 45 min. Recovery occurred under supervision in a warm, quiet environment. ECG data were analyzed using VevoLAB software (version 5.5.0).

### Primary cEC isolation

Primary cECs were isolated from pooled E13.5 *Piezo2*-tdTomato embryos (10–15 hearts per experiment). Atria and outflow tracts were removed, and ventricles mechanically dissociated and filtered through a 70-µm strainer. The retained tissue was digested in Collagenase/Dispase (10269638001, Sigma-Aldrich) for 45 min at 37 °C, homogenized and filtered into a tube with 5 ml DMEM containing 10% FBS (P40-37500, PAN-Biotech) and Gentamicin (G1272, Sigma-Aldrich). An additional 5 ml of the same medium was added, and the mixture was centrifuged for 5 min at 1,200 r.p.m. (160 × *g*).

The pellet was resuspended in 1 ml PBS–BSA 0.5% and centrifuged for 5 min at 300 × *g*. The pellet was then resuspended in 100 µl PBS–BSA 0.5%, and 100 µl of bead–antibody solution was added and carefully homogenized. After 30 min of incubation at RT, 800 µl PBS–BSA 0.5% was added, and bead-bound cells were washed several times with MLEC medium containing 400 ml DMEM-F12 (21041-025, Invitrogen), 20% inactivated FBS, 1% penicillin–streptomycin (15140122, Gibco) and 4 ml ECGS-H (C-30120, Promocell).

Cells were resuspended in 500 µl of MLEC, and each pool was seeded in one well of a 24-well plate coated with 0.5% gelatin (G1393, Sigma-Aldrich). The next day, cells were washed with 1 ml of PBS–BSA 0.5%, and 500 µl of MLEC was added to each well.

After reaching confluency (usually 1 week), the cells were dissociated with trypsin and seeded on pillar arrays for electrophysiology.

For the bead–antibody preparation, per heart pool, 6 µl of sheep anti-rat IgG Dynabeads (11035, Dynal Invitrogen) was placed in 1 ml of PBS–BSA 0.5%, washed several times and resuspended in 6 µl of PBS–BSA 0.5% for each pool. Then, 1.5 µl per pool of VE-cadherin antibody was added to the beads and incubated for 1 h at RT with gentle agitation. After incubation, 100 µl of PBS–BSA 0.5% per pool was added and gently homogenized. Technical replicates were generated as follows: embryonic hearts were collected from 5 pregnant females on 2 separate days, yielding over 50 hearts in total. These were divided into groups of 10–15 hearts to create at least 4 independent cEC preparations. Each preparation was plated in a single well for expansion. After expansion, each well was dissociated and the cells were seeded onto multiple pillar arrays, keeping each preparation separate to maintain independent replicates.

### YS culture

YS cells were collected on ice in plating medium, consisting of DMEM-F12 (Invitrogen) supplemented with 2 μM l-glutamine (Sigma-Aldrich), 8 mg ml^−1^ glucose (Sigma-Aldrich), 200 U ml^−1^ penicillin–streptomycin and 10% fetal horse serum. YS cells were treated with 1 mg ml^−1^ collagenase IV (Sigma-Aldrich) and incubated at 37 °C for 15 min in a water bath. Cells were washed 3× with PBS and incubated with 0.05% trypsin (Invitrogen) for 15 min at 37 °C. Collected tissue was triturated in plating medium using a pipette tip and then centrifuged at 900 r.p.m. (60 × *g*) for 10 min. The pellet was resuspended in 1 ml of plating medium and plated on elastomeric pillar arrays or coverslips pre-coated with 4 μg cm^−2^ laminin (Invitrogen). Cells were cultured overnight, and electrophysiology experiments were performed 18–24 h post-dissection.

YS electrophysiological data were obtained from three separate experiments (cell cultures from embryos isolated from three pregnant females).

### siRNA knockdown

YS cells were cultured overnight on pillar arrays. Subsequently, 24 h after plating, cells were transfected with Accell siRNA and Accell media as per the manufacturer’s protocol (Dharmacon, Horizon) using either non-targeting control siRNA (SMARTpool, D-001960-01-20) or *piezo2*-siRNA (SMARTpool, E-163012-00-0020). Electrophysiological recordings were carried out 96 h post-transfection. Every culture of YS cells was made from at least five different YSs of embryos at stage E13.5 from three different pregnant females. Three independent transfections were performed.

### Preparation of pillar arrays

Pillar arrays were prepared following the established protocol^32^. In summary, silanized negative masters served as templates, which were subsequently coated with polydimethylsiloxane (PDMS) from the syligard 184 silicone elastomer kit (Dow Corning) mixed with a curing agent in a 10:1 ratio (elastomeric base-to-curing agent ratio). The mixture was incubated for 30 min, and glass coverslips were positioned on the top of negative masters containing PDMS, followed by banking for 1 h at 110 °C. Subsequently, pillar arrays were peeled from the negative masters. Each pilus within the array had a radius of 1.79 µm and a length of 5.8 µm. Before cell culture use, the pillar arrays underwent plasma cleaning using a Femto low-pressure plasma system (Deiner Electronic) and were salinized using vapor phase (tridecafluoro-1,1,2,2-tetrahydrooctyl trichlorosilane 97% (AB111444, ABCR GmbH & Co. KG)) for 45 min, followed by a coating with EHS laminin and poly-l-lysine in a 1:1 ratio (v:v) for at least 2 h at 37 °C.

### Pillar array experiments

Whole-cell patch–clamp recordings were conducted on YS and cEC cells (isolated as described above) using borosilicate glass pipettes (Harvard apparatus, 1.17 mm × 0.87 mm) with a resistance of 3–6 MΩ. The pipettes were filled with intracellular solution containing the following (in mM): 110 KCl, 10 NaCl, 1 MgCl_2_, 1 ethylene glycol tetraacetic acid (EGTA) and 10 4-(2-hydroxyethyl)-1-piperazineethanesulfonic acid (HEPES); the pH was adjusted to 7.3 with KOH. The extracellular solution contained the following (in mM): 140 NaCl, 4 KCl, 2 CaCl_2_, 1 MgCl_2_, 4 glucose and 10 HEPES; the pH was adjusted to 7.4 with NaOH. Pipette and membrane capacitances were compensated using the auto-function of Patchmaster (HEKA, Elektronik) while series resistance was compensated to minimize voltage errors. For pillar arrays, currents were recorded at a holding potential of −60 mV, sampled at 10 kHz and filtered at 3 kHz using an EPC-10 USB amplifier and Patchmaster software version 2×91 (HEKA, Elektronik). Data were analyzed with FitMaster, version 2 × 91 (HEKA, Elektronik).

A single pilus was deflected using a heat-polished borosilicate glass pipette driven by an MM3A micromanipulator (Kleindiek Nanotechnik) as previously described in Poole et al.^32^. Pillar deflection stimuli range from 1 nm to 1,000 nm, with larger deflections discarded. For quantification and comparison analysis, data were binned by the magnitude of the stimuli (1–10 nm, 11–50 nm, 51–100 nm, 101–250 nm, 251–500 nm and 501–1,000 nm), and the mean current amplitudes within each bin were calculated for every cell. Deflection-gated currents were classified according to their inactivation kinetics: RA (τ_inact_ < 5 ms), IA (τ_inact_ 5–50 ms) and SA (τ_inact_ > 50 ms).

To calculate pillar deflection, bright-field images (Zeiss 200 inverted microscope) were captured using a ×40 objective and CoolSnapEZ camera (Photometrics) before and after every pillar stimulus. Pillar deflection was determined by comparing the light intensity of the center of each pilus before and after every stimulus using a 2D-Gaussian fit (Igor Software, WaveMetrics).

High-speed pressure clamp experiments (ALA Scientific) were conducted on excised outside-out patches from YS cells. Recording pipettes with a final resistance of 6–8 M were used. Positive-pressure pulses were delivered through the recording pipette. The pressure steps protocol involved a series of stimuli ranging from 10 mm Hg to 150 mm Hg, in 20-mm Hg increments, while maintaining the patch potential at −60 mV. Recording solutions were prepared in symmetrical ionic conditions containing the following (in mM): 140 NaCl, 10 HEPES and 5 EGTA adjusted to pH 7.4 with NaOH.

For both methods, currents and biophysical parameters were analyzed using FitMaster (HEKA, Elektronik).

### Calcium imaging

YS cells were cultured on plating medium (DMEM-F12, Invitrogen) supplemented with 2 μM l-glutamine (Sigma-Aldrich), 8 mg ml^−1^ glucose (Sigma-Aldrich), 200 U ml^−1^ penicillin–streptomycin and 10% fetal horse serum, and then loaded with Cal-520 (5 mM, CAL520 AM, AAT Bioquest). Cells were perfused with extracellular solution (in mM: 140 mM NaCl, 4 mM KCl, 2 mM CaCl_2_, 1 mM MgCl_2_, 4 mM glucose, 10 mM HEPES, adjusted to pH 7.4 with NaOH) at RT. Images were acquired with an Olympus BX51WI microscope equipped with a DG4 (Sutter Instruments) and a CoolSNAP ES camera (Visitron). Image acquisition and analysis were performed using MetaFluor (Molecular Devices). Every image was taken in cycles of 3 s. The baseline of the fluorescence (F0) was established by taking the average of the first 10 cycles. Ionomycin (1 µM) was used to normalize the maximum signal of Cal-520. Data were plotted using the formula Δ*F*/F0 = (*F* *−* F0)/F0.

### scRNA-seq

Gene count matrices from single-cell sequencing from E12, E15, P2 and 8-week-old C57BL/6J animals were generated by Cano et al.^22^ and deposited on the Gene Expression Omnibus repository with accession number GSE223266. Sequence data were mapped to the mouse reference genome (mm10, pre-build references v 2.1.0). Custom code was generated using R to analyze the data and generate plots as described in Cano et al.^22^.

### Statistical analysis

Data were analyzed using GraphPad Prism 9.3.1 and tested for normality. Parametric data were compared using unpaired Student’s *t*-test, and nonparametric data were compared using the Mann–Whitney test. Results are reported as mean ± s.e.m. Significance was defined as *P* < 0.05. Exact *P* values and *n* are provided in the figure legends.

### Reporting summary

Further information on research design is available in the Nature Portfolio Reporting Summary linked to this article.

## Supplementary information

Reporting Summary

Supplementary Video 1 A 3D rendering, segmentation and reconstruction of the *Piezo2*-cre; dtTomato fate-mapped E18.5 heart coronary vasculature, related to Fig. 2a.

Supplementary Video 2 Video recording of the optical view of a representative whole-mount *Piezo2*-cre; tdTomato^+^ E18.5 heart, related to Fig. 2a.

Supplementary Video 3 A 3D rendering, segmentation and reconstruction of individual E18.5 hearts immunolabeled against alpha-smooth muscle (αSMA) with the coronary vasculature reconstructed. Video recording (360° horizontal view) of eight *Piezo2*^*+/+*^ hearts (videos 3–10), related to Fig. 6a.

Supplementary Video 4 A 3D rendering, segmentation and reconstruction of individual E18.5 hearts immunolabeled against alpha-smooth muscle (αSMA) with the coronary vasculature reconstructed. Video recording (360° horizontal view) of eight *Piezo2*^*+/+*^ hearts (videos 3–10), related to Fig. 6a.

Supplementary Video 5 A 3D rendering, segmentation and reconstruction of individual E18.5 hearts immunolabeled against alpha-smooth muscle (αSMA) with the coronary vasculature reconstructed. Video recording (360° horizontal view) of eight *Piezo2*^*+/+*^ hearts (videos 3–10), related to Fig. 6a.

Supplementary Video 6 A 3D rendering, segmentation and reconstruction of individual E18.5 hearts immunolabeled against alpha-smooth muscle (αSMA) with the coronary vasculature reconstructed. Video recording (360° horizontal view) of eight *Piezo2*^*+/+*^ hearts (videos 3–10), related to Fig. 6a.

Supplementary Video 7 A 3D rendering, segmentation and reconstruction of individual E18.5 hearts immunolabeled against alpha-smooth muscle (αSMA) with the coronary vasculature reconstructed. Video recording (360° horizontal view) of eight *Piezo2*^*+/+*^ hearts (videos 3–10), related to Fig. 6a.

Supplementary Video 8 A 3D rendering, segmentation and reconstruction of individual E18.5 hearts immunolabeled against alpha-smooth muscle (αSMA) with the coronary vasculature reconstructed. Video recording (360° horizontal view) of eight *Piezo2*^*+/+*^ hearts (videos 3–10), related to Fig. 6a.

Supplementary Video 9 A 3D rendering, segmentation and reconstruction of individual E18.5 hearts immunolabeled against alpha-smooth muscle (αSMA) with the coronary vasculature reconstructed. Video recording (360° horizontal view) of eight *Piezo2*^*+/+*^ hearts (videos 3–10), related to Fig. 6a.

Supplementary Video 10 A 3D rendering, segmentation and reconstruction of individual E18.5 hearts immunolabeled against alpha-smooth muscle (αSMA) with the coronary vasculature reconstructed. Video recording (360° horizontal view) of eight *Piezo2*^*+/+*^ hearts (videos 3–10), related to Fig. 6a.

Supplementary Video 11 A 3D rendering, segmentation and reconstruction of individual E18.5 hearts immunolabeled against alpha-smooth muscle (αSMA) with the coronary vasculature reconstructed. Video recording (360° horizontal view) of seven *Piezo2*^−^^/^^−^ hearts (videos 11–18), related to Fig. 6a.

Supplementary Video 12 A 3D rendering, segmentation and reconstruction of individual E18.5 hearts immunolabeled against alpha-smooth muscle (αSMA) with the coronary vasculature reconstructed. Video recording (360° horizontal view) of seven *Piezo2*^−^^/^^−^ hearts (videos 11–18), related to Fig. 6a.

Supplementary Video 13 A 3D rendering, segmentation and reconstruction of individual E18.5 hearts immunolabeled against alpha-smooth muscle (αSMA) with the coronary vasculature reconstructed. Video recording (360° horizontal view) of seven *Piezo2*^−^^/^^−^ hearts (videos 11–18), related to Fig. 6a.

Supplementary Video 14 A 3D rendering, segmentation and reconstruction of individual E18.5 hearts immunolabeled against alpha-smooth muscle (αSMA) with the coronary vasculature reconstructed. Video recording (360° horizontal view) of seven *Piezo2*^−^^/^^−^ hearts (videos 11–18), related to Fig. 6a.

Supplementary Video 15 A 3D rendering, segmentation and reconstruction of individual E18.5 hearts immunolabeled against alpha-smooth muscle (αSMA) with the coronary vasculature reconstructed. Video recording (360° horizontal view) of seven *Piezo2*^−^^/^^−^ hearts (videos 11–18), related to Fig. 6a.

Supplementary Video 16 A 3D rendering, segmentation and reconstruction of individual E18.5 hearts immunolabeled against alpha-smooth muscle (αSMA) with the coronary vasculature reconstructed. Video recording (360° horizontal view) of seven *Piezo2*^−^^/^^−^ hearts (videos 11–18), related to Fig. 6a.

Supplementary Video 17 A 3D rendering, segmentation and reconstruction of individual E18.5 hearts immunolabeled against alpha-smooth muscle (αSMA) with the coronary vasculature reconstructed. Video recording (360° horizontal view) of seven *Piezo2*^−^^/^^−^ hearts (videos 11–18), related to Fig. 6a.

Supplementary Video 18 A 3D rendering, segmentation and reconstruction of individual E18.5 hearts immunolabeled against alpha-smooth muscle (αSMA) with the coronary vasculature reconstructed. Video recording (360° horizontal view) of seven *Piezo2*^−^^/^^−^ hearts (videos 11–18), related to Fig. 6a.

Supplementary Video 19 A 3D rendering of *Piezo2*^*+/+*^ and *Piezo2*^*R2756H/R2756H*^ E18.5 hearts (videos 19–31), related to Fig. 7a.

Supplementary Video 20 A 3D rendering of *Piezo2*^*+/+*^ and *Piezo2*^*R2756H/R2756H*^ E18.5 hearts (videos 19–31), related to Fig. 7a.

Supplementary Video 21 A 3D rendering of *Piezo2*^*+/+*^ and *Piezo2*^*R2756H/R2756H*^ E18.5 hearts (videos 19–31), related to Fig. 7a.

Supplementary Video 22 A 3D rendering of *Piezo2*^*+/+*^ and *Piezo2*^*R2756H/R2756H*^ E18.5 hearts (videos 19–31), related to Fig. 7a.

Supplementary Video 23 A 3D rendering of *Piezo2*^*+/+*^ and *Piezo2*^*R2756H/R2756H*^ E18.5 hearts (videos 19–31), related to Fig. 7a.

Supplementary Video 24 A 3D rendering of *Piezo2*^*+/+*^ and *Piezo2*^*R2756H/R2756H*^ E18.5 hearts (videos 19–31), related to Fig. 7a.

Supplementary Video 25 A 3D rendering of *Piezo2*^*+/+*^ and *Piezo2*^*R2756H/R2756H*^ E18.5 hearts (videos 19–31), related to Fig. 7a.

Supplementary Video 26 A 3D rendering of *Piezo2*^*+/+*^ and *Piezo2*^*R2756H/R2756H*^ E18.5 hearts (videos 19–31), related to Fig. 7a.

Supplementary Video 27 A 3D rendering of *Piezo2*^*+/+*^ and *Piezo2*^*R2756H/R2756H*^ E18.5 hearts (videos 19–31), related to Fig. 7a.

Supplementary Video 28 A A 3D rendering of *Piezo2*^*+/+*^ and *Piezo2*^*R2756H/R2756H*^ E18.5 hearts (videos 19–31), related to Fig. 7a.

Supplementary Video 29 A 3D rendering of *Piezo2*^*+/+*^ and *Piezo2*^*R2756H/R2756H*^ E18.5 hearts (videos 19–31), related to Fig. 7a.

Supplementary Video 30 A 3D rendering of *Piezo2*^*+/+*^ and *Piezo2*^*R2756H/R2756H*^ E18.5 hearts (videos 19–31), related to Fig. 7a.

Supplementary Video 31 A 3D rendering of *Piezo2*^*+/+*^ and *Piezo2*^*R2756H/R2756H*^ E18.5 hearts (videos 19–31), related to Fig. 7a.

Supplementary Tables 1–3 Supplementary Tables 1–3 present differential gene expression results from scRNA-seq analysis performed on samples collected at three developmental stages: embryonic day 12.5 (E12.5) and day 15.5 (E15.5), and postnatal day 2 (P2). Each table includes genes with associated statistical metrics including raw and adjusted *P* values, average log_2_(FC) and the proportion of cells expressing each gene in the two cell populations compared (*Piezo2*^+^ and *Piezo2*^−^ cECs). These datasets support the findings shown in Fig. 3d.

## Source data

Source Data Fig. 3 Statistical source data.

Source Data Fig. 4 Statistical source data.

Source Data Fig. 5 Statistical source data.

Source Data Fig. 6 Statistical source data.

Source Data Fig. 7 Statistical source data.

Source Data Extended Data Fig. 4 Statistical source data.

Source Data Extended Data Fig. 5 Statistical source data.

Source Data Extended Data Fig. 7 Statistical source data.

Source Data Extended Data Fig. 9 Statistical source data.

## Data Availability

Data that support the findings of this study are available in the article and Supplementary Information. Gene count matrices from single-cell sequencing from E12, E15, P2 and 8-week-old C57BL/6J animals (mouse reference genome mm10) were generated by Cano et al.^22^ and deposited in the Gene Expression Omnibus repository with accession number GSE223266.

## Code availability

The code used for computer-vision-based 3D coronary artery reconstruction is available on GitHub at https://github.com/Kainmueller-Lab/piezo2_branching_point_analysis. The code has been deposited in a capsule with Code Ocean as well.
