# Supplementary material for: Mechanosensitive PIEZO2 channels shape coronary artery development
Source: Nat Cardiovasc Res. 2025 Jun 27;4(7):921–37. doi: 10.1038/s44161-025-00677-3 (PMC12259469; doi:10.1038/s44161-025-00677-3)
Supplement: Supplementary file 1 — Reporting Summary [file 44161_2025_677_MOESM1_ESM.pdf]

nature portfolio

Corresponding author(s): Annette Hammes

Last updated by author(s): May 7, 2025

# Reporting Summary

Nature Portfolio wishes to improve the reproducibility of the work that we publish. This form provides structure for consistency and transparency in reporting. For further information on Nature Portfolio policies, see our Editorial Policies and the Editorial Policy Checklist.

## Statistics

For all statistical analyses, confirm that the following items are present in the figure legend, table legend, main text, or Methods section.

| n/a | Confirmed | |
|---|---|---|
| ☐ | ☒ | The exact sample size (*n*) for each experimental group/condition, given as a discrete number and unit of measurement |
| ☐ | ☒ | A statement on whether measurements were taken from distinct samples or whether the same sample was measured repeatedly |
| ☐ | ☒ | The statistical test(s) used AND whether they are one- or two-sided<br>*Only common tests should be described solely by name; describe more complex techniques in the Methods section.* |
| ☒ | ☐ | A description of all covariates tested |
| ☐ | ☒ | A description of any assumptions or corrections, such as tests of normality and adjustment for multiple comparisons |
| ☐ | ☒ | A full description of the statistical parameters including central tendency (e.g. means) or other basic estimates (e.g. regression coefficient) AND variation (e.g. standard deviation) or associated estimates of uncertainty (e.g. confidence intervals) |
| ☐ | ☒ | For null hypothesis testing, the test statistic (e.g. $F$, $t$, $r$) with confidence intervals, effect sizes, degrees of freedom and $P$ value noted<br>*Give P values as exact values whenever suitable.* |
| ☒ | ☐ | For Bayesian analysis, information on the choice of priors and Markov chain Monte Carlo settings |
| ☒ | ☐ | For hierarchical and complex designs, identification of the appropriate level for tests and full reporting of outcomes |
| ☒ | ☐ | Estimates of effect sizes (e.g. Cohen's $d$, Pearson's $r$), indicating how they were calculated |

*Our web collection on statistics for biologists contains articles on many of the points above.*

## Software and code

Policy information about availability of computer code

Data collection

Leica MZ 10F stereomicroscope was used with LASX software from Leica Microsystems (Leica, Wetzlar Germany).
Leica DM5000B light microscope with LASX software was used for bright field images.
LSM700 laser scanning microscope was used with the Zen software from Car Zeiss (Zeiss microscopy, Germany) for confocal image acquisition.
Cleared and immunolabelled whole E18.5 hearts were imaged with a Zeiss Lightsheet 7 microscope, with the ZEN 3.1 (black edition) LS software. Post-acquisition processing was performed with the ZEN 3.4 (blue edition) software to fuse the dual-side light sheets of each sample while the FIJI BigStitcher plug-in 112 was used to stitch the tiles.
Echocardiography was performed on the Vevo 3100 ultrasound machine (VisualSonics Fujifilm) together with the MXD550D ultrasonic probe.
Software for Data Acquisition: Vevo LAZR-X (VisualSonics Fujifilm), Version 3.2.6
Patchmaster software (version 1.4.1, HEKA, Elektronik GmbH, Germany) was used to collect patch-clamp recording data.
Pillar deflection was determined by comparing the light intensity of the center of each pilus before and after every stimulus using a 2D-Gaussina fit (Igor Software, WaveMetrics, USA).

Data analysis

For immunohistochemistry on tissue sections and whole mount samples, microscopy images were processed using ImageJ 2.14.0 (Fiji, NIH, Bethesda, Maryland, USA, https://imagej.net/ij/) and IMARIS 10.0 software (Bitplane, Zürich, Switzerland).
Post-acquisition processing for light sheet microscopy images was performed with the ZEN 3.4 (blue edition) software to fuse the dual-side light sheets of each sample while the FIJI BigStitcher plug-in was used to stitch the tiles.
GraphPad Prism 9.3.1 (GraphPad Software Inc, La Jolla California, USA) was used for statistical analyses.
R software 4.3.1 (open source) was used to analyze the scRNAseq data and to generate plots.
VevoLAB (VisualSonics Fujifilm), Version 5.5.0: used for data analysis of echocardiography data.
Pillar deflection was determined by comparing the light intensity of the center of each pilus before and after every stimulus using a 2D-

Gaussina fit (written script for Igor Software, WaveMetrics, USA, Poole K., et al., 2014).
Patchclamp recordings were analyzed using FitMaster (version 2x92; HEKA, Elektronik GmbH, Germany).
The segmentation and 3D reconstruction were used to qualitatively and quantitatively study the coronary architecture.
The plugin Filaments from Imaris were used to measure the ostia to circumflex length.
Calcium imaging: Images were acquired with an Olympus BX51WI microscope equipped with a DG4 (Sutter Instruments) and a CoolSNAP ES camera (Visitron). Images acquisition and analysis were done using MetaFluor® (Molecular Devices).
Computer-vision based 3D coronary artery reconstruction: refined segmentation masks using napari 0.5.6 (https://napari.org/stable/).
We applied binary closing to fill the interior of the blood vessels and skeletonized the resulting 3D masks using Kimimaro (https://github.com/seung-lab/kimimaro). From these skeletons, we computed the distances to each branching point and the total number of branching points. Finally, we manually identified the branching points of interest and recorded their measurements (Ostia-Circumflex). The code is available at https://github.com/Kainmueller-Lab/piezo2_branching_point_analysis and on code ocean.
python 3.9; cloud-volume 12.2.0; h5py 3.13.0; imaris-ims-file-reader 0.1.8; kimimaro 5.0.0; napari 0.5.6; networkx 3.2.1; numpy 2.0.2; pillow 11.2.1; scikit-image 0.24.0; scipy 1.13.1; tifffile 2024.8.30

For manuscripts utilizing custom algorithms or software that are central to the research but not yet described in published literature, software must be made available to editors and reviewers. We strongly encourage code deposition in a community repository (e.g. GitHub). See the Nature Portfolio guidelines for submitting code & software for further information.

## Data

Policy information about availability of data

All manuscripts must include a data availability statement. This statement should provide the following information, where applicable:

- Accession codes, unique identifiers, or web links for publicly available datasets
- A description of any restrictions on data availability
- For clinical datasets or third party data, please ensure that the statement adheres to our policy

All data are available in the main text or the supplementary materials. Gene count matrices from single-cell sequencing from E12, E15, P2 and 8-weeks-old C57Bl/6J animals (mouse reference genome mm10 pre-build references v 2.1.0) were generated by Cano et al. 2024 and deposited on the Gene Expression Omnibus repository with accession number GSE223266. All datasets are mentioned in the data availability section of the manuscript.

## Research involving human participants, their data, or biological material

Policy information about studies with human participants or human data. See also policy information about sex, gender (identity/presentation), and sexual orientation and race, ethnicity and racism.

| | |
|---|---|
| Reporting on sex and gender | n/a |
| Reporting on race, ethnicity, or other socially relevant groupings | n/a |
| Population characteristics | n/a |
| Recruitment | n/a |
| Ethics oversight | n/a |

Note that full information on the approval of the study protocol must also be provided in the manuscript.

# Field-specific reporting

Please select the one below that is the best fit for your research. If you are not sure, read the appropriate sections before making your selection.

☒ Life sciences ☐ Behavioural & social sciences ☐ Ecological, evolutionary & environmental sciences

For a reference copy of the document with all sections, see nature.com/documents/nr-reporting-summary-flat.pdf

# Life sciences study design

All studies must disclose on these points even when the disclosure is negative.

| | |
|---|---|
| Sample size | No statistical methods were used to calculate sample size in advance. Sample sizes were based on prior experience with similar developmental and electrophysiological studies in our lab and in the field, as well as practical considerations including the availability of genetically modified mouse models and the labor-intensive nature of some of the techniques (e.g., light-sheet imaging and in vitro patch-clamp recordings).<br>For morphological analyses, we included up to 8 wild-type and 7 mutant embryos per genotype, which is consistent with or exceeds typical sample sizes used in developmental biology studies to identify robust and reproducible phenotypes. For in vitro experiments (e.g., electrophysiology), at least 3 independent biological replicates were performed, each including multiple cells, ensuring reproducibility across experiments. |

Importantly, the phenotypes observed (e.g., altered coronary artery branching patterns, LV hyperplasia, and distinct electrophysiological currents) were consistent across biological replicates and statistically significant using appropriate statistical tests, supporting the adequacy of our sample sizes for detecting biologically meaningful differences. Full details, including sample sizes and statistical tests, are provided in the figure legends and Methods section.

Data exclusions: No data were excluded

Replication: Biological and technical replicates were performed as stated in the manuscript (Figure legends). All key findings were replicated in at least 3 independent experiments. Morphological phenotypes were observed in multiple independent animals per genotype. Electrophysiological and tube formation assays were repeated with independent cultures or litters, and findings were consistent across replicates.

Randomization: No randomization procedures were applied. Randomization was not applicable to our experimental design because our study primarily involved genetically modified mouse models (Piezo2-Cre;Ai14, Piezo2-/- and Piezo2+/+ mouse embryos, Piezo2 gain-of-function mouse models) at specific developmental stages. All experimental groups were defined by specific genotypes, and comparisons were made between littermate controls and genetically modified animals on C57BL/6N background. Covariates such as developmental stage, and genetic background were controlled by using age-matched littermates of the same inbred strain, and by maintaining consistent housing and experimental conditions. As such, additional covariate adjustments or randomization were not relevant for the aims or methodology of this study.

Blinding: Whole heart light sheet microscopy analyses (coronary artery formation) were blinded considering the genotypes. Blinding was performed during data quantification (e.g., vessel segmentation, coronary artery measurements). Genotype was not revealed to the investigator performing measurements until after analysis was complete.

# Reporting for specific materials, systems and methods

We require information from authors about some types of materials, experimental systems and methods used in many studies. Here, indicate whether each material, system or method listed is relevant to your study. If you are not sure if a list item applies to your research, read the appropriate section before selecting a response.

## Materials & experimental systems

| n/a | Involved in the study | |
|---|---|---|
| ☐ | ☒ | Antibodies |
| ☒ | ☐ | Eukaryotic cell lines |
| ☒ | ☐ | Palaeontology and archaeology |
| ☐ | ☒ | Animals and other organisms |
| ☒ | ☐ | Clinical data |
| ☒ | ☐ | Dual use research of concern |
| ☒ | ☐ | Plants |

## Methods

| n/a | Involved in the study | |
|---|---|---|
| ☒ | ☐ | ChIP-seq |
| ☒ | ☐ | Flow cytometry |
| ☒ | ☐ | MRI-based neuroimaging |

## Antibodies

Antibodies used:
Polyclonal goat anti-PECAM1 (AF3628, R&D Systems, LOT YZU0120101)
Polyclonal rabbit anti-DACH1 (10910-1-AP, Proteintech, LOT 0052490)
Monoclonal rat anti-EMCN (clone V.C7C7, sc-65495, Santacruz, LOT D1117)
Polyclonal rabbit anti-FABP4 (ab13979, Abcam, LOT GR3233572-5)
Polyclonal goat anti-SOX17 (AF1924, R&D Systems, LOT KGA098091)
Polyclonal rabbit anti-RFP (600-401-3979S, Rockland, LOT 800-656-7625)
Monoclonal rat anti-VE-Cadherin (clone 11D4.1, 555289, BD-Pharmigen, LOT 2005276)
Monoclonal mouse anti-SMA (clone 1A4, A5228, Sigma-Aldrich, LOT 0000195447)
Monoclonal mouse anti-SMA-Cy3 (clone 1A4, C6198, Sigma-Aldrich, LOT 0000209582)
Polyclonal Alexa Fluor 488 rat (ab150153 , Abcam, LOT GR3203327-1)
Polyclonal Alexa Fluor 555 rabbit (ab150074, Abcam, LOT GR318110-5)
Polyclonal Alexa Fluor 647 rabbit (ab150075, Abcam, LOT GR312710-4)
Polyclonal Alexa Fluor 647 goat (ab150131, Abcam, LOT GR3246238-3)
Polyclonal Alexa Fluor 647 rat (ab150155, Abcam, LOT GR318807-2)

Validation:
goat anti-PECAM1 (AF3628, R&D Systems): the manufacturer's website states that the antibody was tested in immunohistochemistry, Western blot and flow cytometry. 1) Gao S, Zhou L, Lu J, Fang Y, Wu H, Xu W, Pan Y, Wang J, Wang X, Zhang J, Shao A. Cepharanthine Attenuates Early Brain Injury after Subarachnoid Hemorrhage in Mice via Inhibiting 15-Lipoxygenase-1-Mediated Microglia and Endothelial Cell Ferroptosis. Oxid Med Cell Longev. 2022 Feb 9;2022:4295208. doi: 10.1155/2022/4295208. PMID: 35186185; PMCID: PMC8850040. 2) Yamamoto H, Kon T, Omori Y, Furukawa T. Functional and Evolutionary Diversification of Otx2 and Crx in Vertebrate Retinal Photoreceptor and Bipolar Cell Development. Cell Rep. 2020 Jan 21;30(3):658-671.e5. doi: 10.1016/j.celrep.2019.12.072. PMID: 31968244.
rabbit anti-DACH1 (10910-1-AP, Proteintech): the manufacturer's website states validation in publications: 1) Su T, Stanley G, Sinha R, D'Amato G, Das S, Rhee S, Chang AH, Poduri A, Raftrey B, Dinh TT, Roper WA, Li G, Quinn KE, Caron KM, Wu S, Miquerol L, Butcher EC, Weissman I, Quake S, Red-Horse K. Single-cell analysis of early progenitor cells that build coronary arteries. Nature. 2018 Jul;559(7714):356-362. doi: 10.1038/s41586-018-0288-7. Epub 2018 Jul 4. PMID: 29973725; PMCID: PMC6053322. 2) Chang AH,

Raftrey BC, D'Amato G, Surya VN, Poduri A, Chen HI, Goldstone AB, Woo J, Fuller GG, Dunn AR, Red-Horse K. DACH1 stimulates shear stress-guided endothelial cell migration and coronary artery growth through the CXCL12-CXCR4 signaling axis. Genes Dev. 2017 Jul 1;31(13):1308-1324. doi: 10.1101/gad.301549.117. Epub 2017 Aug 4. PMID: 28779009; PMCID: PMC5580653.
rat anti-EMCN (sc-65495, Santacruz): the manufacturer's website states detection in western blot and the use of the antibody in mouse tissue for immunohistochemistry is referenced in the following citation: Chiang IK, Humphrey D, Mills RJ, Kaltzis P, Pachauri S, Graus M, Saha D, Wu Z, Young P, Sim CB, Davidson T, Hernandez-Garcia A, Shaw CA, Renwick A, Scott DA, Porrello ER, Wong ES, Hudson JE, Red-Horse K, Del Monte-Nieto G, Francois M. Sox7-positive endothelial progenitors establish coronary arteries and govern ventricular compaction. EMBO Rep. 2023 Oct 9;24(10):e55043. doi: 10.15252/embr.202255043. Epub 2023 Aug 8. PMID: 37551717; PMCID: PMC10561369.
rabbit anti-FABP4 (ab13979, Abcam) the manufacturer's website states that the antibody was tested for IHC-P and the use of the antibody in mouse tissue for immunohistochemistry is referenced in the following He L, Tian X, Zhang H, Hu T, Huang X, Zhang L, Wang Z, Zhou B. BAF200 is required for heart morphogenesis and coronary artery development. PLoS One. 2014 Oct 9;9(10):e109493. doi: 10.1371/journal.pone.0109493. PMID: 25299188; PMCID: PMC4192121.
goat anti-SOX17 (AF1924, R&D Systems) the manufacturer's website states that the antibody was tested in western blot and immunohistochemistry. They also provide the following citations: Kanai-Azuma, M. et al. (2002) Development 129:2367. And Katoh, M. et al. (2002) Int. J. Mol. Med. 9:153.
rabbit anti-RFP (600-401-3979S, Rockland) the manufacturer's website states that the antibody was tested in western blot and immunohistochemistry.
rat anti-VE-Cadherin (BD-Pharmigen) the manufacturer website states that is routinely tested via flow cytometry and is reported for immunohistochemistry Vion AC, Perovic T, Petit C, Hollfinger I, Bartels-Klein E, Frampton E, Gordon E, Claesson-Welsh L, Gerhardt H. Endothelial Cell Orientation and Polarity Are Controlled by Shear Stress and VEGF Through Distinct Signaling Pathways. Front Physiol. 2021 Mar 2;11:623769. doi: 10.3389/fphys.2020.623769. PMID: 33737879; PMCID: PMC7960671.
mouse anti-SMA (A5228, Sigma-Aldrich) the manufacturer's website states that the antibody was tested in western blot and immunohistochemistry and provides the following citation for mouse tissue and immunohistochemistry Niedowicz DM, Reeves VL, Platt TL, Kohler K, Beckett TL, Powell DK, Lee TL, Sexton TR, Song ES, Brewer LD, Latimer CS, Kraner SD, Larson KL, Ozcan S, Norris CM, Hersh LB, Porter NM, Wilcock DM, Murphy MP. Obesity and diabetes cause cognitive dysfunction in the absence of accelerated β-amyloid deposition in a novel murine model of mixed or vascular dementia. Acta Neuropathol Commun. 2014 Jun 10;2:64. doi: 10.1186/2051-5960-2-64. PMID: 24916066; PMCID: PMC4229778.
mouse anti-SMA-Cy3 (C6198, Sigma-Aldrich) the manufacturer's website states that the antibody was tested in western blot and mouse wholemount immunohistochemistry.

## Animals and other research organisms

Policy information about studies involving animals; ARRIVE guidelines recommended for reporting animal research, and Sex and Gender in Research

**Laboratory animals**

species: mouse
strain: C57BL6/N (backcross onto C57BL/6N from C57BL/6J for more than 12 generations)
embryonic stages E11.5 - 18.5; adult mice: 10 weeks
genotypes described in the manuscript
housing conditions:
The stocking density of the cages is based on legal requirements and is applied for the following IVC mouse cages: Type II Blue Line with a base area of 536 $cm^2$. The animals are housed at 22 ± 2°C, with a relative humidity of 55 ± 10%, and a 12-hour light / 12-hour dark cycle. The animals are provided with enrichment materials, such as nesting material and hiding places. Mice are fed ad libitum with standard diet and water.

Genetically modified mice:

B6.Cg-Gt(ROSA)26Sortm14(CAG-tdTomato)Hze/J
Ai14 is a Cre reporter tool strain designed to have a loxP-flanked STOP cassette preventing transcription of a CAG promoter-driven red fluorescent protein variant (tdTomato) - all inserted into the Gt(ROSA)26Sor locus. Ai14 mice express robust tdTomato fluorescence following Cre-mediated recombination. Ai14 mice were used at an age of at least 10 weeks for timed matings with Piezo2-cre (> 10 weeks) mice to obtain Piezo2-cre;Ai14 embryos expressing dtTomato in the Piezo2 positive lineage.
B6(SJL)-Piezo2tm1.1(cre)Apat/J
Piezo2-GFP-IRES-Cre knock-in reporter mice express GFP fused to PIEZO2 as well as cre recombinase from the Piezo2 promoter.

B6(SJL)-Piezo2tm2.2Apat/J; EIIa-cre
Exons 43-45 of the Piezo2 gene are flanked by loxP sites in this conditional mutant strain. Cre-mediated excision of the floxed region results in a frameshift mutation that introduces an early stop codon.
Piezo2 constitutive knockout mice:
Generation of Piezo2 null allele (Piezo2-/-) was achieved by crossing with the Ella-cre strain as described in Woo et al., 2014 in Extended data Figure 3 for germline transmission of the recombined Piezo2 allele.

FVB/N-Tg(EIIa-cre)C5379Lmgd/J
This line carries a Cre transgene under the control of the adenovirus EIIa promoter that targets expression of Cre recombinase to the early mouse embryo. Cre-mediated recombination occurs in a wide range of tissues, including the germ cells that transmit the genetic alteration to progeny.

Piezo2R2756H/R2756H:
As described in Sánchez-Carranza, O. et al. Piezo2 voltage-block regulates mechanical pain sensitivity. Brain 147, 3487–3500 (2024)

| | |
|---|---|
| Wild animals | No wild animals were used in this study |
| Reporting on sex | There was no sex discrimination for mouse embryos.<br>For the adult mice, female and male mice were added to the study. |
| Field-collected samples | No field collected samples were used in this study |
| Ethics oversight | Experiments involving animals were performed following the German Animal Protection Act. Procedures involving mice were approved by the Max Delbrück Center for Molecular Medicine (MDC) and the local authorities in Berlin (Lageso). |

Note that full information on the approval of the study protocol must also be provided in the manuscript.

## Plants

| | |
|---|---|
| Seed stocks | *Report on the source of all seed stocks or other plant material used. If applicable, state the seed stock centre and catalogue number. If plant specimens were collected from the field, describe the collection location, date and sampling procedures.* |
| Novel plant genotypes | *Describe the methods by which all novel plant genotypes were produced. This includes those generated by transgenic approaches, gene editing, chemical/radiation-based mutagenesis and hybridization. For transgenic lines, describe the transformation method, the number of independent lines analyzed and the generation upon which experiments were performed. For gene-edited lines, describe the editor used, the endogenous sequence targeted for editing, the targeting guide RNA sequence (if applicable) and how the editor was applied.* |
| Authentication | *Describe any authentication procedures for each seed stock used or novel genotype generated. Describe any experiments used to assess the effect of a mutation and, where applicable, how potential secondary effects (e.g. second site T-DNA insertions, mosiacism, off-target gene editing) were examined.* |
